# Evolutionary Mismatch, Stress, and Competition: Making Sense of Psychosocial Problems in the Polycrisis Era

**DOI:** 10.3390/bs16050650

**Published:** 2026-04-26

**Authors:** Jose C. Yong, Amy J. Lim, Edison Tan, Sarah H. M. Chan

**Affiliations:** 1 School of Social and Health Sciences, James Cook University, Singapore 387380, Singapore; 2 School of Psychology, College of Health and Education, Murdoch University, Singapore 188721, Singapore; 3 School of Social Sciences, Singapore Management University, Singapore 179873, Singapore; 4 Lee Kuan Yew Centre for Innovative Cities, Singapore University of Technology and Design, Singapore 487372, Singapore

**Keywords:** polycrisis, evolutionary mismatch, modern problems, competition, stress, health, wellbeing

## Abstract

Contemporary problems ranging from allergies, myopia, and obesity to chronic anxiety, loneliness, and ultralow fertility can be understood as consequences of evolutionary mismatch intensified by the polycrisis, in which accelerating technological and socioeconomic changes push human adaptations beyond what they evolved to handle. We sought to provide a conceptual review that maps these problems to adaptive needs that are disrupted in highly modernized environments. We then introduce the social evolutionary mismatch and competition hypothesis, which proposes that social aspects of evolutionary mismatch—e.g., increasing population sizes, fragmented communities, rising socioeconomic inequality, constant exposure to inflated social status cues—have a distinct effect of heightening both real and perceived competition. In turn, this perspective can help us make sense of predictable variation in psychosocial outcomes, including obsessive status pursuit, hostility, and social withdrawal. Finally, we outline strategies to lessen the impact of these dynamics by reducing sources of evolutionary mismatch. In sum, we contribute (1) an exposition of how the polycrisis exacerbates evolutionary mismatch and the adaptive needs that are impacted, (2) a theoretical advance identifying mismatch-driven competition as a predictor of multiple problematic outcomes, and (3) a translational framework showing how evolutionary insights can inform interventions to promote well-being in a time of profound societal strain.

## 1. Introduction

The “polycrisis” is a term that has emerged to capture the sense that “disparate crises” like pandemics, geopolitical conflicts, climate emergencies, economic volatility, technological disruptions, and other global problems are interacting “such that the overall impact far exceeds the sum of each part” (World Economic Forum, 2023). Alongside these unfolding events is a growing set of psychosocial problems that has gripped societies worldwide. For instance, reported rates of mental health issues such as mood and personality disorders have risen (Foulkes & Andrews, 2023; D. Richter et al., 2019). Developed nations are experiencing fertility declines which have fallen below replacement levels (Yong et al., 2024), while several developing regions are following suit (Lerch, 2019). Physical health problems such as obesity and diabetes have been described as an escalating global epidemic (Caballero, 2007; Ginter & Simko, 2013), placing mounting strain on public health systems across the world (Abdul Basith Khan et al., 2020; Wellman & Friedberg, 2002). At a psychological and perhaps even existential level, many societies are experiencing a pervasive malaise marked by feelings of loneliness, alienation, insecurity, and cynicism (Newiak, 2024; Parsloe, 2025).

These incidents—from major global shocks to their psychosocial impacts—are increasingly recognized as connected in a manner that mutually reinforces their occurrence (Lawrence et al., 2024; Søgaard Jørgensen et al., 2024). For instance, rising costs of living and widening economic inequality—worsened by financial crises and events like the COVID-19 pandemic—heighten people’s financial and status insecurities, in turn motivating actions that lead to burnout (Petersen, 2020), reduced prosociality (Zhu et al., 2019), and further insecurities, creating feedback loops that intensify societal competitiveness and contribute to economic stagnation or contraction (Lawrence et al., 2024; Yong, 2024). While crucial inroads to improve our understanding of the polycrisis have been made in specialized fields such as psychology (E. P. Richter et al., 2024), sociology (Hurtado Hurtado, 2024), economics (Jacobs, 2024), and politics (Scholtz, 2024), the evolutionary mismatch framework is increasingly recognized as a powerful means of integrating these various perspectives. By focusing on how evolved psychological mechanisms produce maladaptive outcomes in evolutionarily novel environments, this approach helps clarify how individual behaviors and societal conditions interact to produce the psychosocial challenges characteristic of the polycrisis (N. P. Li et al., 2020; Søgaard Jørgensen et al., 2024). However, awareness of this approach remains limited because evolutionary reasoning is not widely understood and its relevance to contemporary problems is underappreciated (Burke, 2014).

In this paper, we aim to shed light on the psychosocial aspects of the polycrisis through an evolutionary mismatch lens. First, we will expand on the evolutionary perspective on human psychology and behavior and next, outline the fundamental human needs that are impacted by modern environments. Through this analysis, and guided by a biopsychosocial model of ecological effects (Engel, 1977), we highlight that evolutionary mismatches tend to increase anxiety and stress especially within a complex of co-occurring shocks, and further propose that the *social* aspects of compounded mismatch have a particular effect of escalating both real and perceived competition. From this hypothesized link between mismatch and competitive stress, a variety of psychosocial outcomes ranging from risk-taking behavior to depression can be accounted for, as well as how these outcomes might predictably vary. Lastly, we demonstrate the practical utility of this framework by suggesting several evolutionarily grounded approaches to cope with these problems. In so doing, we emphasize the need to pay greater attention to evolutionary mismatch in discussions about psychological, social, and institutional challenges during an era of entangled crises.

## 2. Evolutionary Mismatch and Modern Problems

Adaptations are inherited, species-typical traits that develop reliably and have been retained because they helped to solve survival or reproductive problems better than alternative designs during the period in which they evolved (Buss et al., 1998). Our psychology can be understood as consisting of numerous highly specialized, “if–then” information processing mechanisms that take specific environmental cues as input and produce output in the form of thoughts, feelings, and behaviors that enhanced fitness, at least in the ancestral environment under evolutionarily *familiar* conditions (Cosmides & Tooby, 1997). A classic example is our evolved preference for sweet-tasting things, where the if–then rule would be expressed as *if* there is sweet-tasting food (input), *then* eat it immediately (output), which is adaptive in ancestral environments where food was scarce, sweetness indicated calorie-rich sources like ripe fruit, and the urge to eat ensured that valuable sources of nutrition would be consumed before they were gone.

**Figure 1:**
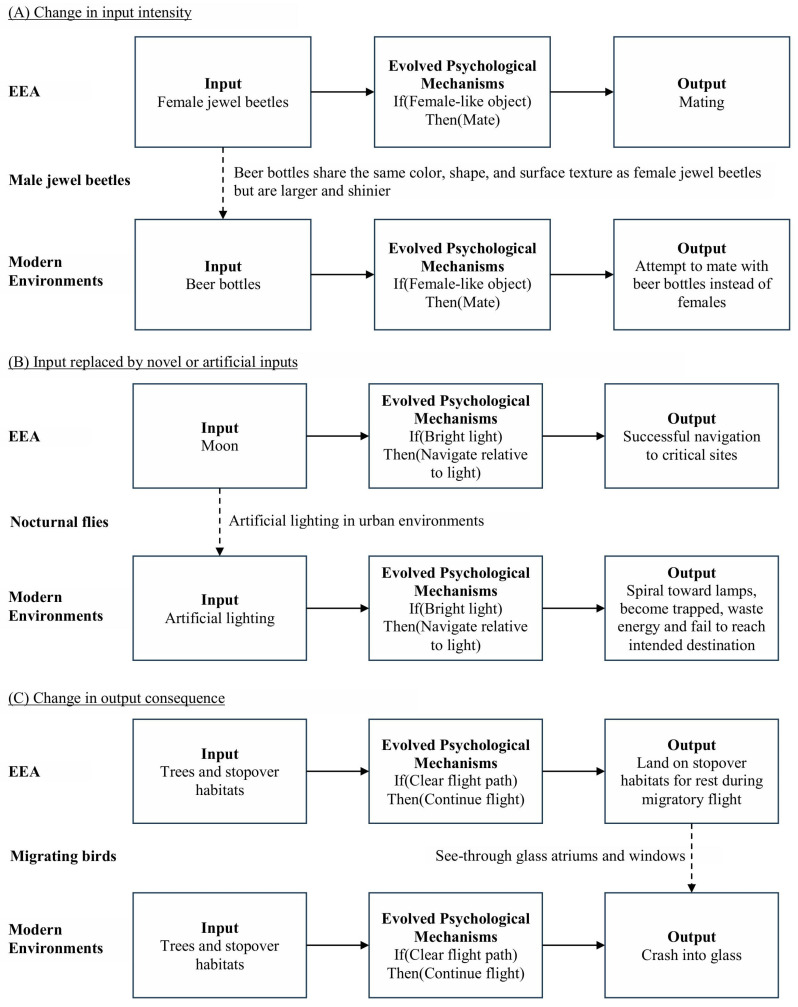
Non-human examples of the three types of evolutionary mismatches.

As adaptive traits typically evolve over thousands of generations, the environment can change faster than the time needed for adaptations to adjust (i.e., adaptive lag), resulting in mismatches between adaptations and evolutionarily *novel* environments (N. P. Li et al., 2018). Problems arising from evolutionary mismatch have been extensively documented across many nonhuman species (Robertson et al., 2013) and can be categorized in three forms (see Figure 1). First, novel environments may contain input cues that differ in intensity from those associated with the ancestral inputs that adaptations were designed for. This can produce hyperstimuli where exaggerated cues dominate over natural ones, such as when male jewel beetles are more attracted to beer bottles than to actual females because the bottles amplify key female features (e.g., color, gloss, texture, size), leading males to preferentially attempt mating with them. Conversely, cues can be dulled, such as when frogs in urban wetlands experience less dense choruses of conspecific calls than in natural breeding ponds, reducing the intensity of acoustic mating cues and impairing reproductive activity. Second, inputs may be replaced altogether, as when artificial lighting substitutes for natural sources of light at night (e.g., the moon), causing nocturnal moths and flies to orient toward and get ensnared by street lamps and building lights. Third, the outputs of otherwise normally functioning adaptive mechanisms may become maladaptive, as when migratory birds (e.g., Catharus thrushes) follow evolved flight behaviors but end up colliding with glass atriums and transparent windows in urban places. In each case, evolutionary novelty in contemporary environments disrupts the effective functioning of evolved adaptations, potentially leading to poorer fitness outcomes.

**Figure 2:**
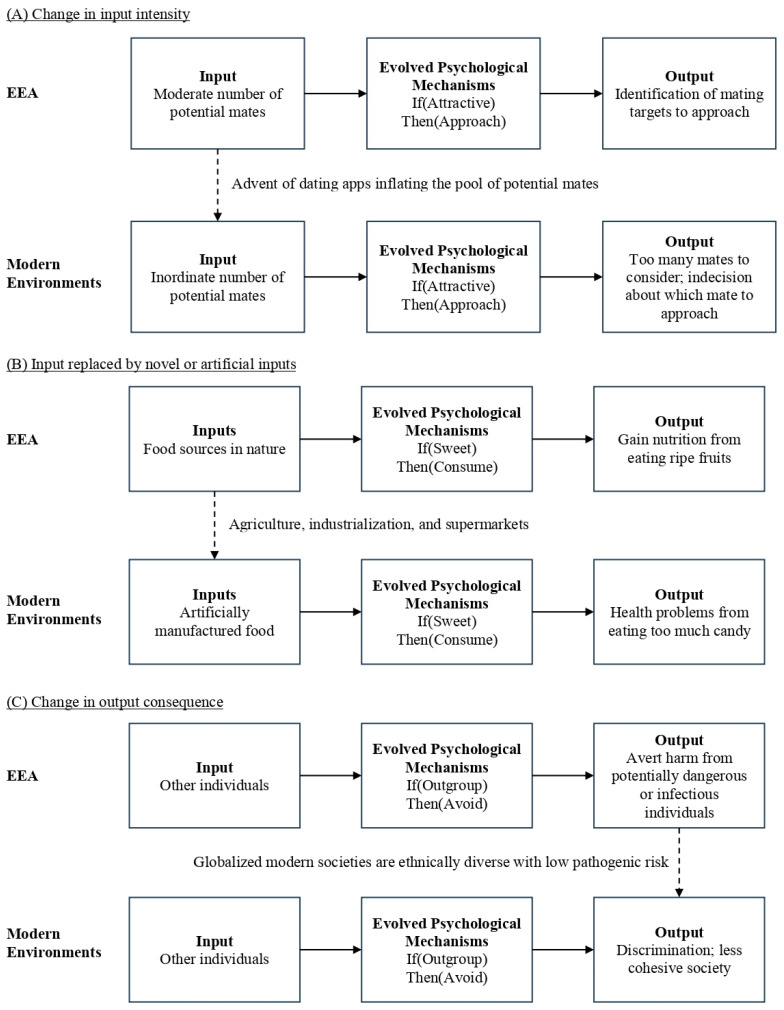
Human examples of the three types of evolutionary mismatches.

These nonhuman cases each have their human analogues (see Figure 2; each case will be discussed more fully in later sections). Indeed, humans are not only vulnerable but also potentially more prone than other animals to evolutionary mismatch due to our tendency to utilize technological and cultural inventions to improve survivability and make life easier (Yong & Kanazawa, 2026). Major revolutions such as agriculture (~12,000 years ago), industry (~250 years ago), and information technology (~50 years ago) have led to significant mismatches between human adaptations and the environment. Considering that even the advent of agriculture was so recent that it constitutes less than 1% of human existence, the technological and cultural changes over the past two centuries will certainly have exacerbated mismatches for our adaptations, which were designed for life in natural settings as nomadic hunter-gatherers in small, kin-based groups. In stark contrast, humans now reside in crowded cities filled with hundreds of thousands of strangers, operate in highly stratified societies, use social media, and lead sedentary lifestyles.

This mismatch lens is instrumental in explaining problems experienced by modern humans. Physiological examples include the aforementioned preference for sweet-tasting things, which motivated consumption in nutritionally scarce ancestral environments but is hijacked today by companies mass-producing unhealthy foods, leading to metabolic and cardiovascular disorders (Gluckman & Hanson, 2006); or modern medicine and hygiene practices which eradicated the microbial inputs that assisted the immunity of early humans, making us susceptible to infections, allergies, and gut issues if those modern practices are not adopted (W. Parker & Ollerton, 2013; Souëf et al., 2000). This logic has also been extrapolated to non-physiological problems, specifically psychosocial ones like social status anxiety (Yong et al., 2024), body image disorder (Yong et al., 2017), loneliness (Newiak, 2024), and more.

### Polycrisis as Amplifier of Evolutionary Mismatch

While modern settings already contain many manmade and artificial features which diverge from the conditions wherein our psychology evolved, a polycrisis environment deepens these mismatches by layering multiple systemic shocks that engage adaptations outside of their functional range (Søgaard Jørgensen et al., 2024). Experts have defined the polycrisis as “the causal entanglement of crises in multiple global systems in ways that significantly degrade humanity’s prospects” (Lawrence et al., 2024, p. 2; Table 1 provides some examples of polycrisis components). Some crises take the form of acute shocks, such as war or economic collapse, whereas others overlap with earlier and more gradual technological and cultural transformations, including consumer capitalism, social media, and demographic change (e.g., overcrowding, expanding migrant populations). As such, it can be difficult to delineate where a long-running transformation ends and crisis begins. For example, the mental health crisis has been linked to the rise of social media more recently as well as to social fragmentation that has been occurring for much longer due to urbanization (Milgram, 1972; Newiak, 2024), while even something more acute like climate disruption reflects, in part, the cumulative consequences of fossil fuel-driven industrialization and capitalistic excesses. For our purposes, we view the polycrisis as encompassing both acute shocks and gradual yet disruptive transformations, particularly when the growing interconnectedness across economic, technological, political, and ecological systems allows impacts in one region or system to spill over into others. This distinguishes the polycrisis from other historical periods of high stress, such as plagues or the World Wars, which were dominated by a comparably smaller number of discrete events rather than ongoing interactions of multiple crises that permeate everyday life.

As these various crises are not just coinciding but causally interacting, their combined impact exceeds what each would produce alone. For example, climate-related disruptions and geopolitical conflict can jointly exacerbate energy and food insecurity, which in turn produces knock-on effects across economic and political systems. Importantly, whereas ancestral stressors were typically localized and contained, contemporary polycrisis dynamics expose individuals to persistent accumulated threats that are simultaneously material and informational in nature. Economic precarity, geopolitical instability, and ecological disruption pose genuine risks to livelihoods and security, while digital media continuously transmit news about these threats and prevent media consumers from disengaging psychologically. Thus, individual-level strain arising from “ordinary” mismatch can be further compounded by polycrisis-related pressures and exposure to persistent threat-related information. Awareness of such evolutionarily novel threats that are global, abstract, and continuous without indications of resolution can in turn trigger sustained and unprecedented levels of chronic anxiety (E. P. Richter et al., 2024) and prompt short-term, defensive strategies that further entrench these problems in the long run. For example, people often respond to insecurity by prioritizing individual or local survival over collective cooperation or global coordination, thereby reinforcing the very insecurity that prompted those responses (Søgaard Jørgensen et al., 2024). To trace how these forces culminate in modern psychosocial problems, we begin by outlining how contemporary environments undermine our ability to satisfy several fundamental adaptive needs in the next section.

## 3. Adaptive Needs Impacted by Evolutionary Mismatch

To be sure, technological and cultural innovations in modern contexts do not always cause problems, at least not initially since they are introduced to address needs and deficiencies. Indeed, the invention of goods and services like vehicles, supermarkets, and the internet helps to solve significant problems such as travel, food procurement, and communication. However, prolonged technological use and cultural immersion creates new problems as the environment becomes increasingly divorced from that in which our ancestors lived, resulting in modern humans leading increasingly unnatural and mismatched lives as more technologies are introduced to deal with those problems (Yong & Kanazawa, 2026). As multiple mismatches accumulate and interact, they reinforce one another to produce broader patterns of systemic misalignment that are increasingly difficult to undo (Søgaard Jørgensen et al., 2024).

It is important not to conflate evolutionary mismatch with general socioecological strain. Unpleasant or aversive experiences like anxiety in response to threat, pain following loss, or stress in competitive contexts can arise under evolutionarily typical conditions and reflect the normal functioning of adaptive systems (Nesse, 2020). By contrast, for an outcome to count as a problem arising from evolutionary mismatch, it must involve conditions that diverge from those under which adaptations were selected and calibrated. Under such conditions, the inputs these adaptations rely on may be absent or encountered at evolutionarily atypical levels, leading mechanisms that would normally guide effective behavior to produce maladaptive outputs because they are misaligned with present demands.

We will first discuss how modern settings with high levels of industrialization, urbanicity, complex economies, technological penetration, and interacting crises interfere with our evolved human nature. We adopt the needs-based approach (Kenrick et al., 2010), which emphasizes how deficits in fitness-relevant domains (e.g., energy, safety, social connection, mating opportunities) define the adaptive problem to be solved and coordinate the mechanisms to address those deficits. Moreover, although evolved mechanisms operate without requiring conscious awareness, they often engage psychological processes that are experienced as needs, urges, and motives (Aunger et al., 2021). Framing adaptive systems in needs-based terms therefore highlights their motivational and experiential dimensions, providing a clearer and more intuitive link between evolutionary function and psychobehavioral outcomes. In the sections that follow, we detail several adaptive needs and how modern conditions create mismatches with the ancestral contexts in which these needs evolved, while reserving discussion of the consequences of these mismatches for the subsequent section. Table 2 summarizes the primary sources of evolutionary mismatch arising from differences between ancestral and modern environments.

### 3.1. Biophilic Needs

To function effectively, our ancestors evolved to be attuned to features of the natural habitats in which they resided, developing physiological capacities to manage exposure to environmental microbes (Souëf et al., 2000) as well as perceptual systems to process ecological cues like diurnal cycles and stimuli found in nature (Gilbert et al., 2023; Júnior et al., 2016). While early humans could not tolerate every natural hazard, they likely possessed extensive knowledge of their surroundings, including local flora, weather patterns, and signs of predators (Acharya, 2011; Salali et al., 2016), which further aided survival as they shared and refined this information within their communities (Hewlett et al., 2024). Over evolutionary time, individuals with an affinity for and skill in engaging with plants, animals, and other elements of nature were more likely to survive and reproduce. E. O. Wilson’s (1984) biophilia hypothesis frames this as an inclination toward nature and life-like processes. Such an orientation would have been adaptive in ancestral contexts where environments rich in familiar ecological cues were linked to safety, whereas environments lacking such cues signaled uncertainty and potential danger.

Much of contemporary life unfolds indoors under artificial lighting and climate control, surrounded by walls, flat surfaces, and uniform textures that provide comfort and safety but lack the rich, multisensory variety of the natural world. Outdoor urban spaces are dominated by synthetic materials, mechanical noise, and repetitive patterns, similarly offering little of the ecological cues that guided perception and behavior in ancestral settings. This sensory deprivation is compounded by the cognitive demands of modern life, which center on multitasking and rapid attention shifting between technologically mediated symbolic stimuli (Loh & Kanai, 2016; Rückriem, 2009). Instead of perceiving and responding to immediate, physical features of the environment, such as movement in the grass or changes in light and wind, modern humans interact with abstract representations like words, numbers, icons, and screens. This shift from a more in-the-moment, natural sensory immersion to symbolic and rapid engagement is more taxing on cognitive resources and, when coupled with impoverished sensory environments both indoors and outdoors, may blunt the biophilic mechanisms that once supported attentional balance, emotional regulation, and physiological restoration (Kaplan & Kaplan, 1989; Ohly et al., 2016).

### 3.2. Disease Avoidance Needs

Infectious outbreaks from the bubonic plague in the 14th century to the 1918 Spanish flu and the most recent COVID-19 pandemic have claimed countless lives and impose significant selection pressure on humans. In response, humans possess adaptations to manage the threat of disease, such as a physiological immune system to neutralize pathogens that have entered the body. However, fighting an ongoing infection is metabolically expensive, so having an additional system that reduces the odds of an infection occurring at all is advantageous. This adaptive need is met through a *behavioral* immune system that facilitates the avoidance of pathogens and other sources of disease (Schaller & Park, 2011). Avoidance responses are activated when disease-connoting cues are perceived, such as coughing and sneezing, foul odors, and skin abnormalities (Ackerman et al., 2009; Miller & Maner, 2012). Because the cost of missing a genuine infection threat is higher than the cost of a false alarm, this system produces an overperception bias that treats even traits denoting poor health (e.g., obesity, old age) as signs of disease (Miller & Maner, 2012).

Several mismatches exist between these disease-avoidance mechanisms and modern environments. First, the behavioral immune system responds to not just actual but also *potential* sources of pathogens. As organisms typically evolve to be resistant to local microbes, non-local individuals are more likely to carry novel pathogens that are dangerous to locals who have not yet adapted to them (Diamond, 1997). Hence, humans may have evolved to be wary of such individuals, which manifests as aversions to unfamiliar persons (Schaller & Park, 2011). Research has shown that people exhibit greater introversion, intolerance, or disgust toward individuals perceived as coming from pathogen-rich ecologies (Faulkner et al., 2004; Mortensen et al., 2010). While such tendencies may have been adaptive in ancestral contexts where people lived in relatively isolated tribes and encounters with outgroups were rare and potentially dangerous, we now inhabit globalized, cosmopolitan societies where ethnic diversity and intergroup interactions are routine. Yet our evolved disease-avoidance mechanisms have not kept pace with these changes, leaving us predisposed to biases against outgroups in relatively benign contexts.

Second, although bacteria, viruses, and parasites can cause disease, they play a role in regulating immune function by engaging pattern-recognition receptors which trigger the release of anti-inflammatory cytokines and promote the development of regulatory lymphocyte populations (Belkaid & Hand, 2014; Mazmanian et al., 2008). Repeated low-dose stimulation of these receptors induces a state of desensitization that dampens inflammatory signaling, thereby limiting excessive autoreactive responses. Modern hygiene practices, however, eliminate many of the microbes and pathogens to which early humans were constantly exposed. While these advances lower infectious disease risk, they also create sterile environments that deprive the immune system of the microbial stimuli it evolved to depend on for proper regulation (Strachan, 1989). By removing these low-level exposures, modern environments limit the immune system’s ability to learn tolerance and maintain balance, which can contribute to dysbiosis and proneness to allergic and autoimmune conditions (Augustine et al., 2022).

### 3.3. Need for Stimulation

Psychologists have long noted that humans have a need for stimulation and sensation seeking (Sales, 1971; Zuckerman et al., 1980). The capacity to find stimulation rewarding is crucial in motivating the search for sensations that increase engagement in adaptive behaviors. For instance, it has been argued that like other animals, humans have basic drives for movement and activity (Rowland, 1998; Stults-Kolehmainen, 2023) which are necessary for exploration, maintaining physical fitness, acquiring desired objects, and escaping from threats. Excessive inactivity results in the activation of drive and prompts urges to satiate it, such as feelings of restlessness accompanied by a desire to move (Stellar, 2012). Likewise, stimulation can be achieved through other sensory modalities including sight, touch, taste, sound, and smell, and humans are typically drawn to pleasant sensations produced by appropriate stimulation in these areas (Berridge & Kringelbach, 2008, 2015). Crucially, these drives operate without requiring awareness of their adaptive outcomes. The pleasure of savoring ripe fruit or encountering something novel ensured nutrient intake and learning, while dopamine release reinforced the behavior (Stellar, 2012). By contrast, deficits in stimulation are experienced as aversive, as is reflected in studies showing that participants prefer receiving electric shocks over having nothing to do (T. D. Wilson et al., 2014).

Our ancestors who lived in grasslands or forest habitats evolved to find nature stimulating and engaging without being too overwhelming. For instance, the fractal-like patterns in natural settings (e.g., plants, landscapes, clouds) possess a level of complexity that is moderately stimulating and yet allows attention to defocus, thus reducing executive function load (Hagerhall et al., 2015). Looking at greenery is commonly suggested as a way to deal with stress (Cytowic, 2016; B. Jiang et al., 2014), and hospital patients recover faster when their wards have windows allowing them to gaze at trees and nature (Hartig et al., 2003). Similarly, activities in nature, such as hiking or human–animal interactions (Chen et al., 2024), would have been sufficiently captivating, and natural foods satisfying to the palate. However, humans today are surrounded by stimuli that are less rich and yet more frequent and intense than those of ancestral environments, such as rectilinear architecture and repetitive textures, mechanical noise from vehicles and electronic devices, and high-contrast signages and rapid image transitions that change hundreds of times per minute. Gustatory and olfactory experiences are also increasingly intensified and standardized through industrial processing and artificial additives. Conversely, environments such as classrooms and offices tend to be uniform and perceptually dull, with activities that are often detached from biologically primary forms of multisensory engagement such as exploration, foraging, or social play and instead involve evolutionarily novel, abstract tasks like reading, calculating, or manipulating symbols on screens (Geary, 1995; Swanepoel et al., 2017). This mismatch between our evolved sensory systems and the modern sensory landscape alters how we experience stimulation and can leave us both over- and under-stimulated (Yong & Kanazawa, 2026).

### 3.4. Need for Certainty

Humans crave certainty, which drives the search for information and resources that make the world feel coherent and predictable (Kossowska et al., 2022; van der Sluis, 2025; Yong et al., 2021b). In harsh ancestral environments where mistakes could be fatal, a bias toward certainty promoted vigilance and behaviors that reduced the likelihood of danger, such as ensuring that a foraging site was free of predators or that a shelter was sturdy and secure. Beyond avoiding immediate threats, the same drive would have supported strategic pursuits by motivating early humans to know where and when certain plants would bear fruit or which hunting grounds would yield better returns, thereby facilitating more efficient planning and allocation of resources and effort. Certainty needs are also reflected in the impulse to secure or stockpile resources like food to preempt future uncertainty about the availability of those resources. This drive is partially mediated by the dopaminergic reward system (Barron et al., 2010; DeYoung, 2013), which kept our ancestors engaged in the relentless pursuit of vital resources and opportunities. Feeling uncertain evokes anxiety (Miceli & Castelfranchi, 2005) which triggers the urge to impose structure on randomness, such as when participants who were primed to feel out of control saw patterns in random dot arrays (Whitson & Galinsky, 2008), deep-sea fishermen engage in more superstitious rituals than their near-shore counterparts (Malinowski, 1948), and day traders resort to pseudoscience in the face of market volatility (Rogelberg, 2024). Patients also experience better recovery outcomes when provided with uncertainty-reducing information (Ng et al., 2022), thus underscoring the psychologically and physiologically restorative effect of certainty.

Modern environments saturated with mass media, consumerism, and technology offer much more information and options than ancestral conditions did. Because humans evolved to attend to adaptively relevant cues when available (B. A. Anderson, 2021), these excesses are difficult to ignore and can overwhelm the cognitive systems designed to filter and prioritize information. When extended to the social realm, humans evolved to live in tightly knit groups where nearly all social events were personally relevant, making us predisposed to treat even trivial social information as important (Yong & Li, 2018). Consequently, networking platforms and modern forms of heightened social connectedness cause people to care about more social information than is necessary or optimal. People who live in large cities or use dating apps are also exposed to an evolutionarily unprecedented number of potential romantic partners, which can impact the ability to make relationship decisions (Apostolou et al., 2024).

Another accelerant of certainty-driven mismatch is quantification through numerical abstraction. While there is evidence that early humans could count based on fossil records of tally sticks that were likely used to track quantities such as trade items or lunar cycles (Marshack, 1972), it wasn’t until around 8000–3000 BCE that numerical abstraction emerged in the form of clay tokens in Mesopotamia (Ifrah, 2000) and a positional number system (base 60) used by the Sumerians for trade, astronomy, and administrative tasks (Schmandt-Besserat, 1996). Formal numeral systems arose from 2000 BCE onwards, including the Indo-Arabic numerals around 500 BCE from which the 0–9 digits we use today originated. With the ability to quantify and compare, humans increasingly faced demands to evaluate and optimize everything from material possessions to social status and potential mates (Espeland & Stevens, 2008). Modern life intensifies this mismatch through metrics such as prices, ratings, likes, and various other statistics, providing levels of abstraction and choice complexity that outstrip the capacities our decision-making systems evolved to manage.

Finally, polycrisis events impose chronically high levels of uncertainty. Simultaneous conflicts such as the Russia–Ukraine war, the Israel–Palestine conflict, and US–China tensions create widespread unpredictability about global security (Elsner et al., 2025), while economic instability through inflation, housing shortages, rapid market fluctuations, and recessions further destabilize people’s expectations about livelihoods and long-term financial planning (Igan, 2024; Sergeyev et al., 2025). In pre-digital societies, awareness of conflicts and economic disruptions occurring elsewhere in the world remained low, but individuals today regularly encounter news about geopolitical escalation, financial turmoil, natural disasters, and social unrest, resulting in a pervasive sense of ambient uncertainty that continually activates evolved mechanisms for threat detection, predictability-seeking, and control (Kesner & Horáček, 2022).

### 3.5. Need for Connectedness and Belonging

People have a need to connect with others and belong in groups because unlike solitary animals, humans are ill-equipped to survive alone. Individually, humans are often defenseless against dangerous predators and unable to withstand harsh environments, but being part of a group provides advantages including shared knowledge, complementary competencies, and strength in numbers (Yong et al., 2021c). Such cooperative and organizational tendencies enabled our ancestors to band together to build shelters, share expertise in dealing with injuries or illnesses, and solve various adaptive challenges. One vital form of cooperation is food sharing, which increases the reliability of food supplies in contexts of variable availability and thus provides greater security than hoarding individually (Plana et al., 2023). Such practices are then reinforced by expectations of reciprocity (Gurven, 2004), which become embedded in collective norms that further strengthen group cohesiveness (Jaeggi & Gurven, 2013). Connectedness and inclusion also impact reproductive success, as women who are more integrated into social networks and hold higher status receive greater alloparental support and have more viable descendants (Bogin et al., 2014; Page et al., 2017).

Social environments have undergone significant changes through modernization. Despite our desire to belong and connect with others, many people now live alone and maintain weaker relationships with family members (O et al., 2021). This shift is partly driven by urbanization as economic opportunities draw people to cities where housing is costly and space is limited, making co-residence with extended kin unfeasible (L. Li & Wu, 2019). Modern labor markets also demand geographic mobility, scattering family members across regions and reducing opportunities for sustained contact (Frankel & DeWit, 1989), while cultural norms that emphasize individual autonomy and independence further reinforce the trend of living apart from kin (Hartanto et al., 2024). Thus, urban environments, while densely populated, are typically composed of weakly tied social networks, resulting in more exposure to and interactions with strangers rather than trusted kin or long-term acquaintances (Delia, 2020).

A related feature of modern life is the rise of social media. Although these platforms are designed to facilitate connection, they reshape social interaction in ways that differ markedly from ancestral forms of bonding (A. J. Lim & Tan, 2024). Online networks often emphasize breadth over depth as the number of acquaintances is extended well beyond the size of social groups humans evolved to manage (Dunbar, 1993). Features such as notifications and algorithmically controlled content feeds alter the dynamics of attention, reciprocity, and reputation maintenance (Brady et al., 2023), leading to social exchanges that are more fragmented and contrived than in-person interactions (Turkle, 2011). Thus, social media reproduces some cues of social inclusion while radically transforming the underlying structure and scale of human connectivity (O et al., 2024).

### 3.6. Self-Esteem Needs

Self-esteem has been argued to be an “overall positive-negative attitude toward the self” (Tafarodi & Swann, 1995, p. 322). In line with this view, the drive to maintain high self-esteem reflects the desire to view oneself positively, which is adaptive in fostering the confidence needed to act effectively in the world (Yong et al., 2021b). However, the observation that humans possess a self-esteem drive sparked the idea of self-esteem as an end in itself, leading to the “self-esteem movement” through the 1980s and 1990s of high self-esteem as a panacea for all of life’s problems. This belief led schools, policymakers, and self-help culture to emphasize boosting self-esteem without regard for underlying causes or mechanisms, resulting in artificially inflated self-esteem which often led to worse outcomes (Baumeister et al., 2003). Researchers seeking to address these issues advanced the sociometer theory of self-esteem, which grounded self-esteem as an adaptive gauge of social inclusion rather than an end in itself (Leary & Baumeister, 2000). In this framework, self-esteem reflects one’s relational value and signals the extent to which an individual is accepted and valued by others. When self-esteem dips through social exclusion or rejection, the sociometer activates compensatory mechanisms aimed at restoring relational value, such as increasing group conformity, interdependence, or prosociality (Leary, 2005; Williams, 2007). Social anxiety is part of this adaptive response by motivating individuals to avoid behaviors that risk embarrassment or ostracism (Maner & Kenrick, 2010).

Large, impersonal societies characterized by rising costs, housing unaffordability, and job precarity alter how individuals evaluate their social and relational value. Many young adults today face barriers to achieving milestones that historically signaled belonging and competence, such as home ownership, stable employment, long-term relationships, and family formation, resulting in traditional pathways to self-worth being less accessible (Fry, 2023). Digital technologies further reconfigure how self-esteem is regulated. Social media platforms expand social comparisons considerably and enable near-constant exposure to others’ curated self-presentations and introduce metrics of social value (e.g., likes, followers, algorithmic visibility) that translate social inclusion into quantifiable feedback (Dinh & Lee, 2025; Sánchez-Hernández et al., 2022). These interactions often emphasize conformity and signaling over the authentic, physically based exchanges typical of offline communities, thereby shifting self-esteem regulation toward more externally mediated forms of validation (A. J. Lim & Tan, 2024).

### 3.7. Need for Status

Being regarded highly carries important benefits, including influence over others and access to resources and social alliances (van Vugt & Tybur, 2015). Individuals with higher status are often treated with deference rather than opposition (Henrich & Gil-White, 2001) and are preferred as allies and mates (Von Rueden et al., 2011). As such, people are motivated to achieve and maintain positions of high or at least adequate status relative to others. The fitness benefits of having status shaped adaptations to monitor and manage standing within groups by attending to cues of dominance, prestige, and relative social position (van Vugt & Tybur, 2015). Individuals quickly gauge status differences even with minimal social cues (Koski et al., 2015) and may act prosocially (Flynn et al., 2006) or aggressively (Buss & Shackelford, 1997) to maintain or enhance social status. Status anxiety motivates individuals to be mindful of status and seek to redress any potential deficits in social standing (Yong et al., 2024).

Ancestral humans lived in kin-based bands of roughly 100–150 people that were highly egalitarian and interdependent (Boehm, 1999; Dunbar, 1993). Members of such groups typically knew each other intimately, and status was based less on material accumulation and more on contributions to the group, such as competence and generosity (Henrich & Gil-White, 2001). Under these conditions, social monitoring mechanisms functioned well as status differences were moderate and reversible through compensatory actions (e.g., increased cooperativeness; Leary, 2005). In contrast, contemporary societies comprise vast, impersonal populations with steep hierarchies and pronounced inequality (Yong et al., 2024). Modern technologies expose individuals to a limitless array of others through media and digital networks, continuously broadcasting cues of prestige, wealth, and social success (A. J. Lim & Tan, 2024). These conditions engage status-monitoring adaptations in unprecedented ways. Whereas status differences in ancestral environments were narrow and could be offset through effort, modern disparities are often extreme and unbridgeable (Yong et al., 2024). In sum, individuals are now encountering levels of inequality and high-status exemplars far beyond those found in ancestral contexts, resulting in an environment saturated with rank and prestige cues that continuously stimulate status-attentive mechanisms originally calibrated for small-scale, egalitarian contexts.

### 3.8. Mating and Reproductive Needs

Although evolution is often described as *survival* of the fittest, organisms that survive but fail to reproduce do not pass on their genes. *Reproduction* is therefore the true “engine of evolution” (Buss et al., 1998, p. 534) and has drawn much attention in evolutionary psychology toward adaptations for sexual selection. Individuals who effectively secured reproductively viable partners and maintained parental partnerships were more likely to raise surviving offspring, thereby passing on the genes that code for those successful mating behaviors (Symons, 1979; Buss & Schmitt, 1993). Mate preferences therefore evolved to guide individuals toward reproductively valuable partners, with sex-differentiated patterns reflecting differences in men’s and women’s fertility spans and parental investment obligations. In particular, men tend to prioritize physical cues of fertility (e.g., youth, sexual maturity), whereas women place greater emphasis on cues of resources and status (N. P. Li et al., 2002). Moreover, because females typically incur high reproductive and parental investment costs, women evolved to be more sexually selective—displaying greater sexual coyness and choosiness in mate selection—which in turn drives men to compete for female choice through qualities signaling competence and willingness to commit (Symons, 1979). These adaptations historically promoted pair bonds that balanced sexual and cooperative goals. Importantly, ancestral mating took place within small, kin-based communities where reputations were transparent and social interdependence was high (Boehm, 1999). Within these contexts, individuals had to acquire mates through socially sanctioned means such as arranged betrothals or demonstrating qualities that fostered long-term commitment (Fletcher et al., 2015). Mating success was thus embedded in cooperative group life, where enduring bonds supported effective parenting and mutual survival.

Contemporary societies feature large populations that expand potential partner pools well past ancestral limits. Where our ancestors encountered only a small, familiar circle of potential mates, modern humans now navigate vast and often impersonal networks in which reputations are opaque and social accountability is weakened (Delia, 2020; Zimbardo, 2007). Large cosmopolitan cities and digital connectivity have intensified social comparison and expose individuals to exaggerated cues of attractiveness, wealth, and success (A. J. Lim & Tan, 2024; Yong et al., 2024). Traits that once signaled reproductive potential are now mimicked or amplified through symbolic displays ranging from conspicuous consumption to carefully selected online personas, thereby weakening their diagnostic value. Modern technologies also accelerate mating decisions as dating apps and digital interactions compress courtship into short cycles of impression formation and matching, prioritizing efficiency and immediacy over the slower trust-building processes that committed relationships require (Degen & Kleeberg-Niepage, 2025; Freyth & Jonason, 2025). The proliferation of sexualized media and platforms (e.g., OnlyFans) further increases the perceived availability and casualization of sexual opportunities. As a result, having strict requirements before sex can occur may come to feel less socially aligned, making women’s evolved preference for selectivity harder to pursue (Anciones Anguita & Checa Romero, 2025; Coyne et al., 2019). Conversely, men face intensified competition as social and mass media extend the field of male competition beyond local communities, pitting men against the most visibly high-status and desirable individuals (Larsen, 2023). The result is a mate selection ecology that operates at a scale, pace, and degree of abstraction far removed from the small, transparent, and socially regulated settings in which human mating psychology evolved.

## 4. Consequences of Evolutionary Mismatch

To make sense of the consequences of the divergences between modern and ancestral environments outlined in the preceding section, we adopt a biopsychosocial framework (Engel, 1977) to describe how adaptations function problematically due to these divergences across physiological, psychological, and social domains. Accordingly, we first examine how evolutionary mismatches interfere with perceptual and cognitive systems, leading to both physical (biological/physiological) and mental (psychological) ill-being. We then highlight that mismatches in the social domain have a unique effect of intensifying competition—both real and perceived—thereby introducing a distinct set of pressures that extend beyond more basic physiological and psychological dysfunction.

### 4.1. Physical Health Problems

Many contemporary physical health issues arise because the inputs that adaptations depend on to regulate bodily functions and stress responses are lacking or excessive in modern contexts, causing them to instead malfunction and produce the following problems.

#### 4.1.1. Diet and Nutrition

In ancestral contexts where nutrition was scarce, individuals with strong preferences for sweet tastes were more likely to eat energy-dense foods (e.g., ripe fruits) whenever they were available, thus motivating the immediate consumption of foods that could not be stored or might be consumed by others. In modern contexts, however, the widespread availability of refined sugars and processed foods misfires this adaptation as the mechanisms that prompt consumption only when high-calorie foods were rare are now continuously activated, resulting in overconsumption and metabolic disorders such as obesity and diabetes (Gluckman & Hanson, 2006). Similarly, ancestral appetites for fats—advantageous when nutritious foods were scarce—now predispose individuals to excessive intake of saturated fats and ultra-processed products, driving dyslipidemia and weight gain (Garnås, 2025; Nouri et al., 2023). Salt-seeking adaptations that helped maintain electrolyte balance also lead to overconsumption today, elevating blood pressure and contributing to global hypertension burdens (Batuman, 2013).

#### 4.1.2. Sedentary Lifestyles

Human physiology evolved under conditions requiring sustained physical activity for foraging, hunting, and nomadic life as adaptive metabolic inputs. The modern sedentary lifestyle driven by structural and technological transformations that minimize the need for movement, including screen-based work, automobile-dependent urban design, domestic convenience technologies (e.g., washing machines, smart-home automation, on-demand delivery services), and digital entertainment that substitutes physical and social activity with virtual engagement, represents a stark input mismatch and predisposes individuals to insulin resistance, obesity, and cardiometabolic disease (Owen et al., 2011). Physical inactivity also impairs innate immune functions such as natural killer cell activity and vaccine responsiveness, thereby heightening vulnerability to infection (Nieman & Wentz, 2019). Large cohort studies further show that prolonged sitting predicts type 2 diabetes and all-cause mortality, even among those who meet recommended exercise guidelines (Patterson et al., 2018).

#### 4.1.3. Microbial Tolerance and Immunity

Modern hygiene, antibiotic use, and indoor living have drastically reduced human exposure to microbes that were once essential inputs for immune system training and homeostasis. This idea is encapsulated in the “hygiene hypothesis” (Strachan, 1989), which posits that although hygiene advances have markedly decreased infectious morbidity, they have coincided with a rise in allergic and autoimmune conditions as reduced microbial exposure disrupts the development of immune tolerance and microbiome diversity. Comparative studies, including cross-cultural and animal samples (Bach, 2018; Haahtela et al., 2015), demonstrate that early-life exposure to diverse microbial and parasitic environments has protective effects. The eradication of helminthic worms, for instance, which co-evolved with early humans to stimulate the production of molecules that tune down the immune system and facilitate microbiome balance, has been identified as a cause of immune dysregulation and chronic inflammatory conditions today (W. Parker & Ollerton, 2013). Consistent with these findings, rates of asthma, atopic dermatitis, and type 1 diabetes have increased over the past four decades even as major infectious diseases have declined (Bach, 2018; Deckers et al., 2012).

#### 4.1.4. Nature Deprivation

Modern urban environments deprive people of the restorative and regulatory effects of nature exposure. According to attention restoration theory, nature elicits “soft fascination”—a gentle form of attention engaged by inherently interesting but non-demanding stimuli like drifting clouds, rustling leaves, flowing streams, or sunrays through tree branches (Kaplan & Kaplan, 1989). These stimuli quietly hold attention while leaving our directed-attention capacity free to rest and recover, resulting in a mind that is engaged but not taxed. When such stimuli are few or replaced by synthetic hyperstimuli in built environments, the directed-attention system can be overstimulated, leading to attentional fatigue, heightened stress, and impaired affective regulation (Pham & Sanocki, 2024). Reduced time outdoors has also been strongly linked to rising global rates of short-sightedness, particularly in urban populations (Dolgin, 2015). Natural outdoor light stimulates retinal dopamine release which inhibits excessive eye elongation, whereas artificial indoor lighting and sustained near-focus tasks (e.g., screens, reading) promote myopic development. People raised in highly urbanized environments also often display biophobia, or discomfort with natural settings and aversion to contact with soil, plants, or animals (Patuano, 2020). Even subtle reductions in natural cues—such as using urban-themed phone wallpapers (Chan et al., 2025)—can weaken people’s sense of connectedness with nature. Chronic disconnection from biodiverse environments is thus associated with reduced microbial exposure, elevated cortisol, increased blood pressure, and poorer eyesight (Dolgin, 2015; Yao et al., 2021), all of which undermine physical and psychological health and general wellbeing.

### 4.2. Mental Health Problems

Recent research has revealed an inverse relationship between evolutionary mismatch—marked by urban crowding, limited nature exposure, physical inactivity, social fragmentation, and pervasive digital engagement—and psychological wellbeing (O et al., 2024), indicating that many contemporary mental health issues stem from evolved mechanisms being maladaptively engaged outside their design context. As modern conditions impose evolutionarily novel demands on systems suited for smaller, more stable social groups and natural environments (O & Brüne, 2019), the following mental health challenges emerge.

#### 4.2.1. Psychological Disorders

Reviews suggest that psychological disorders become more prevalent as societies industrialize and develop (Magomedova & Fatima, 2025), with some researchers calling this a “disease of modernity” (Hidaka, 2012). This trend is consistent with the evolutionary mismatch perspective because developed societies feature rapid socioeconomic change, technological penetration, urbanization, and weakened communal structures, all of which create environments that deviate from those in which human psychology evolved to deal with.

Adopting an evolutionary approach can help to make sense of many psychological disorders at a fundamental level. For example, competitive tendencies that functioned optimally in the smaller groups that typified ancestral tribes are amplified by chronic social comparisons wrought by mass and social media, leading to maladaptive outcomes such as body image concerns and eating disorders in women (Yong et al., 2017) and status-driven stress and excessive risk-taking in men (Pak, 2025; Schmidt et al., 2021). Anxiety disorders reflect threat detection and vigilance systems that are overly activated by ambient and persistent stressors ranging from economic insecurity and continuous performance monitoring to environmental crises and war (Lass-Hennemann et al., 2024; Stein & Nesse, 2011). Insomnia has been attributed to a mismatch between evolved circadian regulation systems and exposure to artificial lighting, screen use, and irregular work schedules, which disrupt natural cues that optimized sleep timing and quality (O et al., 2021). Other conditions—including attention-deficit/hyperactivity disorder (Swanepoel et al., 2017), autism (Bilbo et al., 2012), Tourette’s syndrome (Reser, 2017), gambling addiction (Spinella, 2003), bipolar disorder (Rantala et al., 2021), and schizophrenia (Abed & Abbas, 2011)—have similarly been analyzed from a mismatch perspective. While the extent to which some of these conditions are attributable to mismatch has been debated (Dein, 2024; Hoogland & Ploeger, 2022), we hope to illustrate the potential scope and applicability of evolutionary mismatch by highlighting these cases and invite readers to explore them further.

#### 4.2.2. Overstimulation and Adaptation-Level Escalation

Modern environments inundate individuals with artificial sensory and cognitive stimulation ranging from fluorescent lighting and dense traffic to digital screens and on-demand entertainment, producing concentration difficulties (Cytowic, 2024), anxiety and stress (W. Xu et al., 2024), and sleep disturbances (O et al., 2021). Activities that unnaturally overactivate the brain’s reward circuitry, such as synthetic drug use, hyperpalatable foods, online gaming, and other forms of digital media engagement, can trigger compulsive behaviors and addiction (Kuss & Griffiths, 2012; Passeri et al., 2023). In these technologically saturated environments, people struggle to downregulate attention to constant stimuli, leading to habituation processes described by adaptation-level theory (Helson, 1964). Because individuals evaluate incoming stimuli relative to prior experiences, thresholds for satisfaction continually rise. As initial pleasures fade, stronger or more frequent stimulation is required to achieve the same reward, generating cycles of craving and dissatisfaction (Edwards, 2018).

This escalation also explains why those accustomed to constant modern entertainment feel restless without their screen devices and find natural experiences underwhelming. The perpetual pursuit of novelty and intensity fosters chronic boredom, which many seek to conveniently alleviate through “cheap” forms of stimulation such as smartphones, social media, and streaming services (Tam & Inzlicht, 2024). Such boredom may also influence health behaviors, such as bedtime procrastination and poorer sleep quality (Teoh et al., 2021). Yet boredom may have evolved as an adaptive signal to search for meaningful engagement or creative exploration when stimulation is lacking (Park et al., 2019; Bench & Lench, 2013). When quickly numbed by trivial digital or consumer distractions, this motivational function is blunted, reducing valuable opportunities for creativity and learning. Dependence on these easy rewards not only dulls sensitivity to subtler, naturally rewarding experiences but also increases risks of screen addiction (Seaward, 2020), cognitive decline (Malaei, 2024), and poorer general health (Cytowic, 2024; Rojas-Carvajal et al., 2022).

#### 4.2.3. Information Abundance and Cognitive Overload

Modern environments expose humans to unprecedented amounts of information, options, and quantifiable metrics via mass media, consumerism, and digital technology. This creates cognitive strain through information overload, where decision quality declines because data exceed processing capacity (Eppler & Mengis, 2004), and the paradox of choice, where too many options induce indecision and dissatisfaction (Schwartz, 2004). Evolved needs for certainty and control compound these effects, prompting individuals to engage in excessive information-seeking and optimization attempts. Such tendencies can manifest as hoarding (Wheaton & Topilow, 2020), over-analyzing trivial choices (Schwartz, 2004), or obsessive self-monitoring (Levesque, 2011). Once humans gained the ability to quantify value, these drives expanded into metric fixation as people track everything from their weight and income to followers and time with obsessive precision (A. J. Lim & Tan, 2024).

The quantification of time usefully illustrates how this fixation is problematic at a mundane level. Where people once waited patiently for a bus to arrive, real-time tracking through apps like Google Maps now encourages constant monitoring, micro-timing departures, and fretting over minutes lost. This technological precision, while useful for improving efficiency and coordination, also heightens time-related stress and erodes the patience previously sustained by the acceptance of uncertainty as an unavoidable feature of life (Rosa, 2013). Because these systems make life more convenient and predictable, people find it difficult to forgo them despite their psychological costs (Yong & Kanazawa, 2026). Meanwhile, cultural pressures for efficiency and productivity further intensify mental fatigue as individuals feel compelled to answer emails during commutes, track steps while walking, or listen to “productive” podcasts during rest in order to optimize every waking hour, leaving little room for psychological recovery and fostering burnout as the lines between work and leisure blur (Rosa, 2013). The constant influx of information about economic volatility and global crises through algorithmic systems also keeps people in a state of hypervigilance, perpetuating a cycle of monitoring that maintains stress responses in a constantly activated and mentally exhausting state (Peckham, 2023).

#### 4.2.4. Social Disconnection and Superficial Connectedness

Paradoxically, even as modern individuals live amid larger populations than ever before, loneliness is rife as people are increasingly surrounded by strangers, resulting in more abundant yet shallow ties (Newiak, 2024). These transient and transactional interactions wrought by urban anonymity and high mobility may fail to satisfy evolved needs for belonging and trust, thereby limiting opportunities for enduring relationships and collective identity while cultivating a pervasive sense of isolation. Digital communication intensifies this paradox as social media platforms designed to simulate connection hijack affiliative drives for attention and approval, producing intermittent social rewards (e.g., likes, followers, notifications) that magnify comparison, envy, and fear of exclusion (A. J. Lim & Tan, 2024; Groenestein et al., 2024). Avid users may expend increasing effort maintaining an online presence and social relevance but gain little genuine intimacy in return, thus overburdening cognitive limits on meaningful relationships as authentic interactions are replaced with performative ones (Turkle, 2011). Modern social environments thus exploit evolved social motives while depriving individuals of the connection and communal stability those motivations functioned to secure, leading to poorer mental and subjective wellbeing (O et al., 2024).

#### 4.2.5. Online Validation and Fragile Self-Worth

Social media has also become an avenue for fast and constant social validation. For instance, individuals may prioritize posting curated or attention-seeking content (e.g., clickbait or ragebait posts), engaging in viral challenges (e.g., ice bucket challenge, mannequin challenge), or signaling ideological alignment (e.g., hashtags like #metoo or #blacklivesmatter) to receive quick acknowledgment, rather than investing in contributions or accomplishments with real or enduring value. This dynamic is intensified as young adults increasingly turn to online spaces to affirm their relational value, particularly as traditional sources of self-esteem (e.g., stable employment, home ownership, life achievement milestones) become less accessible. Heavy engagement with social media approval metrics can lead to inauthentic self-presentation through pressure to comply with a variety of demands and result in poorer mental health outcomes (Wright et al., 2018). The availability of approval metrics can also reshape how people gauge their own worth. For example, a professional might announce a career milestone on LinkedIn and fixate on how few likes or congratulatory comments they received, thus triggering feelings of inadequacy despite the achievement being objectively significant. These dynamics can foster poorer self-esteem (A. J. Lim & Tan, 2024), chronic anxiety about social exclusion (Groenestein et al., 2024), and the development of false selves (Wright et al., 2018) as modern individuals get caught between the need to feel valued and the fear of being left out.

### 4.3. Intensified Competition and Psychosocial Problems

Beyond general stress, we suggest that the social aspects of evolutionary mismatch exert a distinct effect of intensifying both actual and imagined competition. Several features of modern life converge to produce this outcome. First, the scale and density of contemporary populations exceed those of the small, kin-based bands in which ancestral humans lived, increasing the number of rivals for resources, social attention, and reproductive opportunities (Otterbring et al., 2021; Sng et al., 2017). Second, rapid mobility and urbanization weaken cooperative ties and reciprocity norms, thus diminishing access to reliable, long-term alliances while encouraging more self-protective and individualistic strategies (Newiak, 2024). Third, technologically mediated comparison exaggerates competition psychologically by providing constant, salient cues about peers’ achievements, attractiveness, and social standing (Cytowic, 2024; A. J. Lim & Tan, 2024). Finally, the socioeconomic aspects of the polycrisis—including economic uncertainty, housing scarcity, social inequality, and intensified mate competition—alter the availability and distribution of vital resources and opportunities, rendering success simultaneously more difficult to achieve and yet more consequential (Becker & Murphy, 2009). These converging pressures manifest in multiple outcomes linked to intensified competition.

#### 4.3.1. Costly Signaling

Evolutionarily, humans can attain social status through two main routes, namely *dominance* where deference is enforced via strength, coercion, or fear, and *prestige* where deference is voluntarily granted to those displaying respectable qualities like competence or seniority (Maner, 2017). In modern consumer societies, however, the acquisition of material possessions has become a prominent alternative avenue to demonstrate social standing by functioning as a costly signal through conspicuous consumption (Otterbring et al., 2021). Evolutionary mismatches in status-monitoring mechanisms contribute to elevated materialism in the modern era, as widespread exposure to high-status individuals and intensified status competition compel many to “keep up with the Joneses”. Compared with earlier generations, Millennials and Gen Z’ers place greater importance on goals related to money and image (Pattuglia & Amoroso, 2023; Talwar, 2024). Permissive attitudes toward debt predict luxury purchases and reflect a willingness to borrow in order to signal status when direct acquisition is not possible—a phenomenon described as “going broke to look rich” (Yong, 2024). Similarly, the rise of luxury fashion rental services provides consumers with affordable access to high-end goods while preserving the appearance of status (Ruan et al., 2022). Individuals who are especially motivated to signal status may even rent the interior of a private jet to stage photographs for social media (J. C. Lim, 2023), simulating affluence without actually possessing it.

#### 4.3.2. Elevated Risk Appetite

Intensified competition in modern environments can cause individuals to perceive their current standing as insufficient, in turn motivating actions to gain an edge in competing for status (Yong et al., 2024). Traditional paths to upward mobility may seem too slow or inaccessible, and when faced with rising inflation and costs of living, risk-taking becomes appealing because it offers an opportunity to attain status that would otherwise remain out of reach, even when potential losses are substantial (Kish-Gephart, 2017; Ritterman Weintraub et al., 2015). This dynamic is exemplified by the growing popularity of highly speculative financial instruments such as cryptocurrencies, forex day trading, and “meme stocks” (Aslan et al., 2023; Whybrow et al., 2025). The internet has drastically lowered barriers to entry as trading was once the domain of professionals with institutional access, but now anyone with internet access and a smartphone can easily participate. For many individuals seeking quick wealth without the expertise or resources to trade effectively, such speculation functions less as informed investment and more as a form of legitimized gambling (Aslan et al., 2023). Modern contexts also magnify competition in domains such as occupational prestige (Yong et al., 2019) and social media influence (A. J. Lim & Tan, 2024), where rewards are both highly visible and disproportionately distributed. Competitive industries like technology and finance are often glorified, reinforcing the perception that high risks are justified by the potential for high rewards in income and recognition (Ho, 2009; Satell, 2016). These winner-takes-all settings further incentivize risk-taking, particularly when individuals believe that only bold moves can secure or maintain respectable social standing.

Risk-taking also functions as costly signaling, especially among men (Baker & Maner, 2009). More specifically, the willingness to take risks can act as a signal of confidence and capability, as only those with sufficient competence or resources are presumed able to do so. Yet in modern settings characterized by urban anonymity and digitally mediated signaling, the link between risky behavior and genuine ability is often muddled (Donath, 2007; Milgram, 1972). Men may therefore exaggerate their income or job status (Epstein, 2007) and feel compelled to pursue risky strategies to appear desirable in the dating market (Whybrow et al., 2025). Women may also resort to risky behaviors such as extreme dieting, cosmetic surgery, or reckless sexual behavior to access high-status partners (Blake & Brooks, 2019), particularly during economic downturns (Hill et al., 2012). Digital environments have further extended these dynamics. Because algorithms favor sensational or extreme content, content creators are motivated to take conspicuous risks such as dangerous stunts, public provocations, or intimate self-disclosures to gain visibility and social rewards. Since viral success can translate into career-making sponsorships or follower-based income, people engage in an escalating arms race of ever riskier behaviors to achieve or sustain digital status, though these performances may carry lasting personal costs including reputational damage or regret over actions taken in pursuit of short-term visibility (Cornell & Peden, 2025; Gleeson, 2025).

#### 4.3.3. Low Fertility

Global fertility has declined such that the majority of the human population now lives in countries with fertility rates below the replacement level of 2.1 children per woman (United Nations Department of Economic and Social Affairs, 2024). According to resource-competition models of life history theory (Sng et al., 2017), fertility is affected by the availability of resources and the intensity of competition for those resources. When there are ample opportunities to obtain resources and competition for those resources is low, organisms tend to focus on having more offspring sooner to capitalize on available resources and opportunities, but when resources are scarce and competition is intense, organisms delay reproduction to focus on building individual capacity so as to compete more effectively for resources. In modern cities, dense populations and exposure to large numbers of unrelated individuals create persistent cues of competition while contemporary levels of inequality heighten perceptions of resource scarcity, causing people to delay reproduction until they feel capable enough for parenthood through education, career advancement, and wealth accumulation (Sng et al., 2017; Yong et al., 2025). Our psychology, which evolved in modest tribal settings where status cues were limited, is also constantly exposed to exaggerated wealth and success displays particularly through mass and social media, leading people to underestimate their social position and prioritize making up for imagined status deficiencies over mating and reproductive pursuits (Yong et al., 2024). As such, fertility rates are lowest in industrialized societies where achievement, wealth, and social mobility are highly valued and media technologies are prevalent. These factors are especially pronounced in developed East Asian societies, resulting in trends such as “education fever” which magnify status anxiety to such an extent that many have opted to forgo marriage and childbearing altogether (T. Anderson & Kohler, 2013).

Urban environments and digital dating platforms also interfere with mechanisms for forming and maintaining secure romantic bonds. Choice overload from the abundance of romantic options fosters indecision and unrealistic expectations, while constant exposure to alternative partners reduces satisfaction and commitment toward ongoing relationships (Apostolou et al., 2024). Modern cues also cause evolved mate preferences to function suboptimally. The male preference for physical attractiveness is accentuated by visual platforms like Tinder and Instagram, which prioritize superficial appearance and skew perceptions of population-level attractiveness (Fioravanti et al., 2022), while the salience of sexual opportunities created by sexualized media feeds on men’s proclivities for casual sexual variety (Symons, 1979) and erodes motivations for long-term mating effort (Wisman & Thomas, 2023). Women’s preference for high-status partners and men’s drive to display such status also add to the already highly competitive, achievement-oriented atmosphere of modern settings (Yong et al., 2024).

By removing the constraints that historically moderated mate choice (e.g., limited partner pools, greater effort needed for courtship to proceed), online platforms allow mating preferences to manifest at their logical extremes. Dating apps expose people to global “leaderboards” of attractiveness and desirability, rendering indicators of mate value (e.g., social status, bodily attractiveness) highly salient and compressing millions of potential partners into a single ranked comparison set. However, men and women respond to this ranked environment differently. Women’s evolved selectivity concentrates attention on a small set of highly desirable men, leaving the vast majority overlooked (Neyt et al., 2019). Men, by contrast, evolved to be more accepting of a wider range of partners so long as minimum attractiveness thresholds are met (N. P. Li et al., 2002), and thus tend to direct attention broadly while still targeting at least moderately attractive women. As a result, attractive women receive disproportionate attention while less attractive women receive less, and men must send large numbers of messages to secure even minimal reciprocation (Bruch & Newman, 2018; Topinkova & Diviak, 2025).

These asymmetries result in large-scale hypergamy as many women are drawn to the same few top-tier men, who—facing abundant alternatives—experience reduced incentives to commit (Larsen, 2023). The remaining majority of men and the least attractive women must then either compete intensely for limited attention or withdraw from the mating market altogether. Other downstream effects include reduced relationship stability (Sharabi & Dorrance-Hall, 2024), the normalization of low-commitment interaction patterns (e.g., ghosting, situationships), and the emergence of marginalized subpopulations (e.g., incels; Costello et al., 2024), all of which have a negative bearing on fertility rates.

#### 4.3.4. Disengagement and Apathy

When traditional milestones become unreachable, individuals can experience a threat to self-esteem as pathways to demonstrating social value disappear (Höpfner & Keith, 2021). Many adapt by lowering their aspirations or rejecting conventional success narratives altogether (Crocker & Park, 2003). In China, the *tang ping* (“lying flat”) and *bai lan* (“let it rot”) movements valorize disengagement from relentless competition as a means of psychological self-preservation (J. Xu & Wu, 2025). Similarly, in Western economies, “quiet quitting”—doing one’s job without exceeding expectations—reflects a comparable retreat from occupational striving (Scheyett, 2023). Recent surveys report that only 31% of American employees felt engaged at work—the lowest in a decade—with steepest declines among those under 35 (Harter, 2025). As growing numbers of young adults do not believe that working hard will pay off and view long-term financial planning as futile, they instead prefer spending on hedonic experiences and more immediate needs (Credit Karma, 2025). The collective impact of these coping strategies has broader socioeconomic implications as employers increasingly struggle to elicit labor motivation through conventional incentives (Saraiva & Nogueiro, 2025). A pervasive sense of apathy has also emerged as younger generations are increasingly expressing disinterest in roles and customs that are foundational to societal functioning (e.g., marriage, parenthood), often interpreting them as sources of stress rather than fulfillment (Breeze & Calvert, 2024; K. Parker & Minkin, 2023). Trends such as Japan’s *hikikomori* and Korea’s *honjok* illustrate a more extreme preference for withdrawal and emotional safety over collective participation (M. Jiang, 2025), with evidence showing that these behaviors are emerging in other cultures too (Lin et al., 2022). Overall, these phenomena reflect resigned detachment that adaptively functions as self-esteem preservation in a time of diminishing returns on effort.

#### 4.3.5. Distrust and Cynicism

Opportunities for familiar, repeated interactions in small, kin-based ancestral communities enabled reputations and trust to be established for early humans, rendering long-term cooperation possible and more rewarding than opportunistic behavior (Mesoudi & Jensen, 2012). Modern societies are instead characterized by transient, anonymous, and technologically mediated relationships in which interactions are brief, transactional, and often competitive. Competition places people in a mindset that frames others as rivals and prioritizes selfish and advantage-seeking behaviors over cooperation (Barker & Barclay, 2016; Schurr & Ritov, 2016). When individuals rarely encounter the same people twice and face regular competition for limited opportunities and resources, it becomes adaptive to assume that others are self-interested than to risk being exploited (Balliet & Van Lange, 2013).

While self-protective, habitual distrust limits the capacity to foster interpersonal bonds and can culminate in cynicism—a defensive adaptation to environments where sincerity and reciprocity feel uncertain and the trustworthiness of others is hard to gauge (Turner & Valentine, 2001; Vice, 2011). In such conditions, kindness can be met with suspicion while altruism is interpreted as image management or manipulation. For instance, online displays of charity or activism can be dismissed as virtue signaling, and public expressions of optimism are treated with irony or sarcasm (Brathwaite & DeAndrea, 2025; Kreuz, 2020). Experimental research has also shown that highly cynical individuals tend to respond to threatening social climates with lower empathy and reduced prosocial behavior (Choy et al., 2021). Cynicism extends beyond interpersonal dynamics to broader institutions. High-profile scandals such as corporate fraud (e.g., Enron, Wirecard) and political corruption (e.g., misuse of campaign funds, opaque lobbying) make it harder to assume that institutional systems will act in good faith (Sancak & Loew, 2022; Solé-Ollé & Sorribas-Navarro, 2018). The perception that traditional investments of effort (e.g., building a career, remaining loyal to employers, saving for the future) no longer guarantee stability or upward mobility further erodes confidence in the social contract (Kalleberg, 2012). Even newer systems that claim to subvert institutional corruption, such as the cryptocurrency market, often replicate it as founders enrich themselves while extolling decentralization, and investors rush in less out of belief and more out of fear of missing out. The prevailing sentiment is that only the naïve play fair when everyone else is taking their slice of the pie, which makes collective effort and shared progress more difficult to achieve (Olson, 1965).

#### 4.3.6. Hostility and Aggression

While apathy and cynicism represent inward-facing responses marked by disengagement and withdrawal, a more outwardly directed consequence of a distrusting and ultra-competitive environment is hostility and aggression. Ancestral humans were naturally wary of potentially hostile outgroups, so mechanisms for coalition detection based on appearance and behavioral cues enabled individuals to locate the safety of their ingroup and avoid dangerous outsiders (Kurzban et al., 2001; Miller et al., 2012). Hostility was a natural consequence of this vigilance, as it was safer to respond with caution or antagonism toward unfamiliar individuals than to be welcoming or friendly. Although modern societies are considerably diverse and intergroup interactions can be supported by civic norms (Marschall & Stolle, 2004; van Leeuwen, 2010), hostility can still emerge from high levels of crowdedness, inequality, and distrust as competitive pressures and social faultlines dominate (Jackson & Esses, 2000; Kramer, 2009; Zhang et al., 2025). Indeed, studies have shown that crowding intensifies negative affect and antisocial behavior (Zhang et al., 2023; Zlutnick & Altman, 1972), and urban residents are less likely to help strangers (Korte & Kerr, 1975). Pathogen-avoidance mechanisms add to this problem as the tendency to find unfamiliar groups aversive or disgusting, while adaptive in ancestral contexts where intergroup mixing carried significant pathogen risk (Schaller & Park, 2011), contributes to heightened discrimination and xenophobia in today’s globalized world. Elevated disease salience such as during the COVID-19 pandemic predicted greater social distancing from ethnic outgroups (Meleady et al., 2021), illustrating how an evolved system for pathogen defense can exacerbate modern prejudice.

From an evolutionary standpoint, aggression is an adaptive response to resource and status contests, enabling individuals to co-opt others’ resources, defend their own, deter exploitation, and negotiate hierarchical position (Buss & Shackelford, 1997). Ancestral humans who effectively deployed aggression (or even just its threat) were more likely to secure access to resources, mates, and protection from potential outgroup threats. Studies have shown that aggression increases in contexts of insecurity and competition over limited resources. For instance, communities facing chronic resource constraints display elevated levels of aggressiveness and intergroup conflict (Ember & Ember, 1992), while income inequality predicts higher rates of violent crime (M. Wilson & Daly, 1997). Another psychological mechanism underlying aggression is anger, which is typically triggered when individuals perceive their welfare to be undervalued or that norms of fairness are violated (Sell et al., 2009). Perceptions of unfairness are heightened in modern contexts of pronounced inequality, where resources appear unfairly distributed and the odds seem stacked against some individuals, resulting in increased anger and aggressive behaviors as people seek to compel others to respect their interests and treat them more fairly (Kassab et al., 2025). Anger can also be stoked by rhetoric that frames certain ethnic groups as harmful and threatening. During COVID-19, for instance, Asians were portrayed as disease carriers with terms such as “China virus” and “kung flu”. While this increased both pathogen avoidance and anger, which in turn predicted greater discrimination against them, only anger predicted increased aggression (A. J. Y. Lim et al., under review). This highlights the close link between anger and aggression and emphasizes that periods of intensified stress can escalate outgroup negativity, with salient events such as COVID-19 providing a pretext for the expression of pre-existing hostility.

#### 4.3.7. Depression and Suicidality

Finally, depression and suicidality may emerge when individuals perceive competition as insurmountable and are unable to find ways to preserve self-worth or restore social standing. The social competition hypothesis (Sloman et al., 2003) posits that depression arises from defeat and subordination in social hierarchies. When the pursuit or defense of social status fails, individuals may enter a state of “entrapped defeat”, characterized by feelings of failure, inferiority, powerlessness, and hopelessness. This state reflects a neurobehavioral mechanism to inhibit futile struggle, conserve energy, and reduce the risk of further harm. Complementary accounts include the analytical rumination hypothesis (Andrews & Thomson, 2009), which proposes that depression reallocates cognitive resources toward sustained problem-solving, while the bargaining model (Hagen, 2003) conceptualizes depressive symptoms as a costly signal to motivate others to provide support. In ancestral contexts, temporary activation of these mechanisms may have been adaptive, manifesting as mild, temporary depression or dysphoria to facilitate disengagement, help-seeking, and recalibration of strategies. Depressive episodes are therefore often time-limited and naturally resolve within a period of 3 months to a year (Coryell et al., 1994).

However, in modern environments where competitive failure can feel inescapable, this depressive mechanism can take the form of prolonged clinical depression. Depression rates have been documented to be rising globally (Hidaka, 2012), particularly in stressfully competitive urban environments where individuals feel incapable of meeting social and economic expectations while lacking supportive kin networks (Campos, 1996; Sloman et al., 2003). Exposure to others’ successes and idealized lives on social media further expands competition beyond one’s immediate social group (A. J. Lim & Tan, 2024). Moreover, whereas earlier generations looked up to celebrities who remained socially distant and symbolically distinct, today’s influencers inhabit a psychologically proximate and intimate space with their fans (Gräve, 2017). Their perceived accessibility and reciprocal engagement blur the line between admiration and comparison, as influencers appear not as distant idols but as relatable peers—close enough to make their success feel attainable and obligatory, which can foster subtle yet persistent feelings of inadequacy and the compulsion to keep pace (Pedalino & Camerini, 2022). The erosion of connectedness with nature—once a source of sensory grounding and emotional regulation—may further compound these problems as people prefer to occupy their time in virtual rather than natural environments (Michaelson et al., 2020).

When taken to the extreme, depressive withdrawal can lead to suicidal ideation or behavior. Traditional psychological perspectives often interpret suicide as an attempt to escape unbearable psychological pain, whereas evolutionary accounts offer different functional interpretations (O, 2018). The bargaining or “cry-for-help” model views suicidal behavior as an intensely costly signal designed to elicit aid from those whose support matters for resolving a perceived uncontrollable problem (Syme et al., 2016). Another account, the inclusive fitness model (deCatanzaro, 1980), argues that suicidal thoughts may arise when individuals judge themselves to be burdensome and believe their continued survival would impose fitness costs on close kin. Both accounts hinge on a sense of powerlessness or lack of control over vital outcomes (O, 2018). From a mismatch perspective, strategies that may have been adaptive in kin-dense ancestral environments can dangerously malfunction in stressful modern contexts where people often live far from biological relatives and cries for help may go unnoticed, producing self-destructive inclinations that poorly serve fitness.

## 5. The Social Evolutionary Mismatch and Competition Hypothesis

Based on the consequences described in the preceding section, we advance the social evolutionary mismatch and competition hypothesis (SEMCH) to claim more precisely that social evolutionary mismatch produces heightened competition as a primary emergent outcome. Accordingly, while non-social forms of mismatch (e.g., leading a sedentary lifestyle in a concrete environment surrounded by artificial sounds and lighting) can undermine individual wellbeing through problems such as attentional fatigue (Pham & Sanocki, 2024), poorer health (Patterson et al., 2018), or disrupted sleep (O et al., 2021), they do not inherently generate competitive stress. By contrast, social evolutionary mismatches intensify competition that is both objective, as when more individuals vie for limited status positions (Yong et al., 2024) or desirable partners (Topinkova & Diviak, 2025), or perceived, as when constant exposure to curated content on digital platforms inflates the success or desirability of others (A. J. Lim & Tan, 2024). From the perspective of the SEMCH, competition is not an incidental byproduct but the central pathway through which evolutionarily novel forms of social input translate into downstream psychobehavioral consequences.

### 5.1. Specificity of Social Evolutionary Mismatch and Competition

The SEMCH yields several predictions based on the claim that social evolutionary mismatch preferentially elicits competition. First, evolutionarily novel social inputs (e.g., high population density, high inequality, social media) should more strongly predict competition appraisals, whereas non-social inputs (e.g., absence of nature, built environments) should more strongly predict general stress, cognitive fatigue, and other physiological problems. While existing evidence is broadly consistent with this pattern (Kaplan & Kaplan, 1989; Sng et al., 2017; Ulrich, 1983; Yong et al., 2024), these domains have rarely been examined jointly. Urban environments offer a useful testing context because they contain evolutionarily novel non-social and social inputs simultaneously. Second, the SEMCH discriminately predicts that exposure to evolutionarily novel social inputs should more reliably activate competition-related cognitions, motivations, and behaviors (e.g., competitiveness, selfishness, status vigilance, social comparison, zero-sum thinking, strategic self-presentation) than non-competitive ones (e.g., cooperation, trust, altruism, prosociality).

The SEMCH also clarifies how particular inputs exert their effects. A common oversimplification is the belief that reducing complete exposure to social media or screen devices will reduce negative outcomes such as poorer self-evaluation or life satisfaction (Hartanto et al., 2021). The SEMCH instead emphasizes that targeting the precise input is more critical. For example, individuals more frequently exposed to status-relevant cues through large comparison networks in which displays of attractiveness or success are paired with visible indicators of popularity (e.g., likes, follower counts) should experience greater competitive stress than those who spend similar amounts of time online but encounter fewer status-relevant signals. Consistent with this, prior work shows that the relationship between social media use and self-evaluation depends on the size of social networks (A. J. Lim et al., 2021), and that popularity metrics (e.g., likes) specifically worsen negative affect, particularly when such metrics are publicly displayed (Wallace & Buil, 2021).

### 5.2. Competition-Intensifying Mechanisms of Social Evolutionary Mismatch

We suggest that social evolutionary mismatches can be differentiated into three mechanisms through which modern settings and polycrisis conditions bias adaptive systems toward competitive processing. First, *scale calibration* mismatch arises when social evaluative systems calibrated for small, kin-based groups operate under evolutionarily novel conditions of vastly expanded social networks comprising weakly tied, heterogeneous non-kin members (A. J. Lim et al., 2021). On the one hand, mechanisms adapted for local rank may process population-level information as locally relevant, effectively increasing the number of perceived rivals as extreme exemplars whom one would not normally compare with become salient. On the other hand, exposure to large numbers of socially distant and unfamiliar others may reduce the salience of kinship and cooperative ties, weakening expectations of reciprocity and increasing baseline perceptions of social threat. This mechanism can be tested by manipulating the scope of comparison and the familiarity of the social environment. For example, individuals asked to evaluate their standing relative to their local community should exhibit weaker associations between perceived inequality and competitive stress than those comparing themselves to broader groups that include highly successful or extreme exemplars. Environments framed as containing more unfamiliar individuals (e.g., outgroups) should also reduce perceived social support and increase competitive threat appraisals relative to environments composed of familiar others.

Second, *signal validity* mismatch arises when status cues that were reliable in ancestral contexts because they were costly, difficult to fake, and tightly coupled to underlying traits are replaced by low-cost, selectively curated, and exaggerated cues in modern environments (Yong & Kanazawa, 2026). When such cues are treated as reliable despite their reduced diagnosticity, perceived status differences become distorted. The validity of status information can be experimentally manipulated to compare responses to profiles containing superficial or incomplete cues versus more comprehensive indicators, or by varying whether individuals evaluate their standing using information that presents only top-performing peers versus the full range of peer performance. A “signal inflation” mechanism can also be tested by observing whether inequality predicts perceived competition more strongly when amplified-status cues are salient, such as under conditions of high exposure to curated success signals (e.g., frequenting social influencer profiles) or prominent status markers (e.g., visible luxury goods, public rankings, follower or reputation metrics).

Third, *exposure duration* mismatch arises because social evaluations in ancestral contexts were typically episodic and context-bound, but modern individuals are often continuously exposed to social information without opportunities for disengagement (A. J. Lim & Tan, 2024). Systems adapted for intermittent activation then become chronically engaged. This mechanism can be tested by manipulating the temporal distribution of social information while holding total exposure constant. For example, participants can be exposed to identical social comparison stimuli either continuously within one prolonged session or intermittently across multiple shorter sessions, and differences in perceived competition, competitive stress, or physiological markers of arousal (e.g., heart rate) can be assessed. Organizations or schools can also serve as field settings to examine these effects by comparing environments that provide real-time, continuously updated performance information versus those that provide periodic summary feedback, and assessing whether continuous exposure produces stronger and more persistent competitive responses.

Crucially, the SEMCH emphasizes that it is not the mere presence of social input that is problematic—since competitiveness is one among many adaptively appropriate responses to socially important informational cues—but rather evolutionarily novel configurations of social input that prompt adaptive mechanisms to produce excessive or misdirected competitive responses in modern settings. The effects of social evolutionary mismatch are also likely to vary across individuals differing in susceptibility to evolutionarily novel social inputs. For example, adolescents with less mature self-regulatory systems (Crone & Konijn, 2018), individuals high in sensitivity to social threat (Cools et al., 2005), and people living in societies with high inequality (Rakesh et al., 2025) or economic insecurity (Yong et al., 2021a) might exhibit greater responsiveness to social evolutionary mismatch. Conversely, some people may be less prone to these effects. For instance, the savannah-IQ hypothesis suggests that more intelligent individuals can handle evolutionary novelty better than less intelligent individuals and, thus, may be less negatively impacted by evolutionarily novel social inputs like high population density (N. P. Li & Kanazawa, 2016). Finally, responses to perceived competition can generate self-reinforcing dynamics, whereby strategies to cope with competition themselves create a more competitive climate (Garcia et al., 2020) and render reversals of competitiveness difficult. Taken together, the SEMCH stresses that social evolutionary mismatch does not simply increase the amount of social input but reorganizes it in ways that increase sensitivity to relative standing, social comparisons, and resource and status threats, biasing responses toward competitive behavior at the expense of cooperation or other prosocial orientations.

### 5.3. Downstream Responses to Mismatch-Intensified Competition

**Figure 3:**
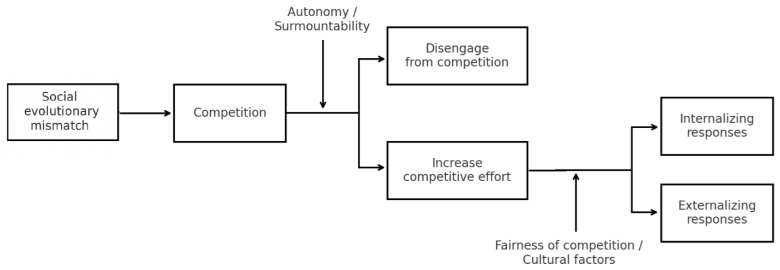
The competition-intensifying effect of social evolutionary mismatch and the various downstream consequences.

The SEMCH can be further expanded to specify how the consequences of mismatch-intensified competition are expected to systematically vary. Figure 3 provides an illustrative overview. Thus far, we have expounded on the first part—social evolutionary mismatch → intensified competition—and listed a wide range of responses to intensified competition. Nonetheless, their occurrence is unlikely to be uniform across individuals and contexts. We suggest that how people respond to competition depends on whether the competition is perceived as *surmountable* and whether they feel *autonomous* in pursuing it (Deci & Ryan, 1985; Randez & Hélie, 2024).

People’s appraisals of the surmountability of competition are shaped by a set of interacting factors including opportunity affordances (e.g., socioeconomic mobility, resource availability; Corak, 2013), institutional trust and fairness perceptions (e.g., whether systems are seen as biased; Thibaut & Walker, 1975), local norms and reference groups (e.g., what levels of success are considered attainable within one’s comparison environment; Huguet et al., 2009), and individual-level factors such as prior success, self-efficacy (Bandura, 1997), and access to social or economic capital (Bourdieu, 1986). People’s sense of autonomy can be shaped by cultural expectations (e.g., norms around failure and success; Wong & Liem, 2023), familial pressures (e.g., parental aspirations, inherited responsibilities; Fuligni, 1999), and economic demands (e.g., financial constraints; Sen, 1999). Together, these factors influence whether competitive environments are perceived as navigable or impossible to overcome, and whether people feel free to decide if they want to continue striving competitively.

When competition is judged to be insurmountable *and* individuals are autonomous to choose what to pursue, they may *disengage* from competition as a protective strategy to preserve self-worth (Crocker & Park, 2003). Such voluntary disengagement represents an adaptive withdrawal from unattainable or coercive goals, allowing individuals to maintain a positive sense of self at the expense of social status or conventional success. Here, we predict that people will valorize less competitive or non-status-linked pursuits, such as prioritizing personal wellbeing over achievement, rejecting long-term career sacrifices, and elevating the ideals of singlehood over the stresses of initiating or maintaining romantic relationships. Apathy or indifference towards traditional markers of achievement and fulfillment may accompany these disengagement attitudes and behaviors.

However, when competition is perceived as potentially surmountable and individuals lack autonomy to withdraw, they are expected to *increase* competitive effort. Behaviors that reflect increased effort include costly signaling and elevated risk appetites, especially when competition is perceived to be so fierce that conventional levels of effort are seen as insufficient. Increased competitiveness is likely to be accompanied by reduced prosociality in the form of attitudes such as distrust and cynicism as well as behaviors such as hostility and aggression.

Success from increased effort—the ideal outcome—would likely reinforce engagement and restore self-efficacy and self-worth (Crocker & Park, 2003). However, and more importantly from our interest in mismatch-related difficulties, failure despite one’s best efforts yields divergent *internalizing* or *externalizing* outcomes (Achenbach et al., 2016) depending on the perceived *fairness* of the competition. If the competition is regarded as fair, failure will likely be accepted and *internalized*, producing responses that are directed inward rather than overtly expressed to others, such as low self-esteem, depression, or suicidality. In contrast, if the competition is perceived as unjust or biased toward some privileged individuals over others, failure is more likely to be *externalized*, manifesting as aggression against others in a system seen as unfairly standing in the way of one’s interests (Sell et al., 2009).

Cultural contexts may also shape how these responses manifest. In *shame-oriented* cultures that emphasize social harmony and collective reputation, failure tends to be experienced not just as a personal shortcoming but as letting others down (Liyanage & Usoof-Thowfeek, 2023), making failure more likely to be *internalized* and result in depression and social withdrawal. Similarly, in cultures that are ethnically or socially homogeneous, individuals are more likely to perceive others as kin or in-group members, so self-directed responses are adaptive in minimizing costs to others and preserving group cohesion in line with inclusive fitness principles (Smith, 2020; Sznycer et al., 2012). Extreme expressions of such withdrawal-oriented coping include social isolation (Heinrichs et al., 2006) or even suicide, where self-elimination functions to avert shame or burden to one’s social group (deCatanzaro, 1980).

In contrast, in more *heterogeneous* or weakly integrated societies where shared identity and kin-like obligations are diffuse, people may feel less inhibited from directing frustration outward against others (Amin, 2025; Arbatli et al., 2020). Under such conditions, competitive failure is more likely to be *externalized*, producing aggression or hostility toward perceived rivals or oppressive societal structures (Blake & Brooks, 2019). Outwardly directed acts of violence can thus be interpreted as retaliatory attempts to redress perceived systemic unfairness or exclusion (McCauley & Moskalenko, 2008). Contemporary manifestations might include disaffected groups such as incels and individuals who commit acts of public violence as expressions of grievance against a social order they perceive as impossible to succeed within.

### 5.4. Stress Test of the Social Evolutionary Mismatch and Competition Hypothesis

The explanatory leverage of the SEMCH can be illustrated by applying it to areas for which other models have offered explanations and showing how it affords incremental predictions. We consider two such competition-related phenomena—low fertility and social status anxiety—and describe how incorporating social evolutionary mismatch yields insights beyond conventional accounts.

#### 5.4.1. Low Fertility

Declines in fertility are commonly explained by economic and sociological accounts that emphasize rising childrearing costs, increased female education and labor force participation, urbanization, and changing family norms (Adserà, 2004; Ogawa et al., 2015). Related psychological accounts including life history approaches suggest that individuals delay reproduction under conditions of uncertainty or resource scarcity in order to invest in status, skill development, or resource acquisition (Sng et al., 2017). Under evolutionarily familiar conditions, this trade-off is adaptive because investments in education, career advancement, or social positioning should enhance future mating prospects and parental capacity, thereby facilitating reproductive success ultimately.

The SEMCH qualifies the predictions derived from these various models. First, the SEMCH predicts that fertility intentions are shaped not only by objective constraints (e.g., income, housing), but also by the structure of social evaluation and comparison environments. Individuals exposed to broader and more extreme comparison sets (e.g., highly curated portrayals of parenting standards or partner desirability) should exhibit lower fertility intentions than those evaluating themselves within more locally bounded contexts, even when objective resources are similar. Likewise, the relationship between socioeconomic status and fertility should be moderated by exposure to amplified social inputs, such that individuals with sufficient resources to pursue mating or reproduction still avoid doing so because awareness of high-status exemplars inflates thresholds for what constitutes “adequate” partnership (Bruch & Newman, 2018; Kenrick & Gutierres, 1980) or parenting (Lee et al., 2025). Finally, the SEMCH predicts that modern conditions will decouple the pursuit of status from its adaptive reproductive function, such that the life history trade-off becomes dysfunctional. Under evolutionarily novel levels of intensified competition, individuals may continue to invest in status beyond the point at which it meaningfully improves mating or parenting outcomes (Yong et al., 2024). As status pursuit becomes self-perpetuating rather than instrumentally linked to reproduction, preoccupation with further status gains may lead people to delay or forego fertility despite having adequate resources or opportunities.

#### 5.4.2. Social Status Anxiety

Social status anxiety has been widely studied in relation to inequality, relative deprivation, and social comparison processes (Wilkinson & Pickett, 2009; L. Xu & Li, 2024). The status anxiety hypothesis predicts that individuals should experience anxiety when they perceive themselves as lower in rank or at risk of downward mobility, particularly in societies characterized by high social disparity or strong emphasis on achievement (Wilkinson & Pickett, 2009). The SEMCH does not challenge these basic associations but advances the more specific claim that modern environments transform status evaluations in ways that profoundly influence the link between inequality and status anxiety. Indeed, research has shown that people draw on multiple and shifting reference groups rather than relying on a single, clearly defined comparison set, complicating status anxiety hypothesis predictions based on objective rank (Yngwe et al., 2003). Other studies have also found that feelings of inferiority and status anxiety do not consistently align with objective socioeconomic position, suggesting that psychosocial responses to inequality depend on more nuanced factors (Layte & Whelan, 2014). The SEMCH provides a more precise account of this variability by proposing that modern environments expose individuals to expanded and heterogeneous comparison contexts, loosening the coupling between objective position and perceived standing as mechanisms adapted for small-scale social ecologies operate over a broader and less clearly bounded set of comparison targets.

Thus, while the SEMCH aligns with the status anxiety hypothesis that high inequality sustains status anxiety by increasing the salience of status differences and maintaining individuals in a state of ongoing social evaluation, it generates at least two differentiating predictions. First, status anxiety should be influenced not only by one’s position within a bounded social group, but also by the scale and composition of comparison environments. For example, status anxiety should arise even in contexts where individuals occupy objectively adequate or even advantaged positions due to comparisons with socially distant but extreme targets. Second, the effects of inequality should depend on how status differences are rendered salient within these environments rather than on inequality alone. Environments that exaggerate status signals should produce greater anxiety than environments with comparable levels of inequality but less salient or more representative status information. Evolutionarily novel social inputs may therefore intensify and prolong status-related preoccupations, making them more pervasive and less easily resolved than would be expected from mere objective inequality.

## 6. Potential Solutions According to the Evolutionary Mismatch Perspective

By clarifying which fundamental needs are misaligned and affected, the current perspective offers a powerful basis for proposing solutions that target the root causes of modern problems rather than simply treating surface-level symptoms (N. P. Li et al., 2020). A key insight is that if these challenges stem from evolutionary mismatch, then efforts should focus on reducing the degree of mismatch wherever feasible. Our proposed interventions begin with more socially oriented evolutionary mismatch-reduction strategies (6.1. to 6.4.) followed by non-socially oriented ones (6.5 to 6.8) and can take two forms. First, interventions can seek to reduce evolutionary mismatch by bringing environmental inputs closer to the conditions in which adaptive systems evolved. Second, where such realignment is impractical, interventions instead aim to mitigate downstream social stress and competition-related responses without altering mismatched environmental conditions. This distinction is critical because many features of modern environments in which the polycrisis is situated (e.g., urban density, digital connectivity, economic structures) are difficult to reverse, necessitating complementary approaches that buffer their psychological consequences. As some scholars argue that a degree of entrapment or irreversibility may even be inevitable (Søgaard Jørgensen et al., 2024), a focus on coping is therefore more pragmatic. Some proposed interventions include the following.

### 6.1. Reducing Perceptions of Crowdedness

Population densities in modern settings are unprecedentedly high, particularly in urban centers around the world where crowding shows few signs of abating. Exposure to such evolutionarily novel levels of crowdedness can elicit heightened anxiety and competitiveness as individuals feel they lack personal space and must vie with others for limited resources (Zlutnick & Altman, 1972). A straightforward solution would be for urban developers to plan at the outset strategies to reduce the concentrated proximity of people within cities, such as by spacing buildings and neighborhoods in accordance with optimal community sizes (Mathur, 2005).

However, altering the structure of preexisting megacities like Tokyo, London, or New York would require such an incredible amount of resources and social engineering that it would be effectively unfeasible. Moreover, modern economies require a large, diverse labor force (Syrett & Sepulveda, 2025), so simply downsizing the population would also be impractical. Recent research showing that people’s sense of crowdedness depends not only on actual or objective population density but also on how their environment subjectively makes them feel—whether they experience spatial restriction or the intrusive presence of others (E. Tan et al., under review)—suggests that interventions should include calibrating the perceptual cues associated with crowding. For instance, urban design that improves the quality of public transport and incorporates natural elements (e.g., parks, greenery), restorative spaces, and noise reduction features can enhance the feeling of open, breathable space, reduce perceived social presence, and enhance connectedness to nature (Patuano, 2020; Srinivasan et al., 2003; Takano et al., 2002). These measures can foster a sense of spaciousness and autonomy even in compact, fixed areas and facilitate the deactivation of evolved stress responses triggered by modern levels of social proximity (Wen et al., 2020).

### 6.2. Expanding the Range of Valued Occupational and Societal Roles

Studies suggest that perceived affordances—people’s subjective sense of what opportunities are available in their environment—play a crucial role in motivating the pursuit of adaptive goals (Diekman et al., 2020; Gibson, 1979). When people perceive viable routes to status (e.g., occupations, wealth) as scarce or highly competitive, they experience status insecurity and become more preoccupied with pursuing these routes, which then creates the effect of making status even less attainable (Yong et al., 2019). For instance, individuals from cultures that obsess over social status may converge on a narrow set of prestigious roles (e.g., medicine, law); this crowding elevates competition and raises barriers to entry (e.g., higher qualifications or more years of working experience required), thereby increasing the perceived value of those roles and further intensifying people’s pursuit of them. Such dynamics carry broad costs including heightened stress, poorer wellbeing, and reduced fertility, as individuals devote disproportionate time and energy to pursuing extended education, overworking to secure promotions, or sacrificing health and relationships in service of status-seeking (Yong et al., 2024).

Increasing the perceived availability and diversity of valued societal roles may therefore attenuate status anxiety and reduce the zero-sum logic that underlies status-conscious engagement with work, relationships, and future planning. Indeed, policies that enhance perceived affordances have already been discussed in relation to fertility, such as supportive family policies, affordable childcare, social safety nets, and conferring greater recognition and social value on parenthood, which can help to reduce stress while strengthening motivations to start and raise a family (Rovny, 2011). By extension, broadening the range of niches through which people can contribute to society, feel valued, and gain dignity—whether via occupations, volunteering, creative pursuits, or civic roles—can relieve compulsive orientations toward status competition (Rappaport, 2002; Yong et al., 2019). Practical approaches include elevating the prestige and compensation of lower-status occupations and promoting the importance of prosocial or non-market activities (e.g., volunteering). By expanding the set of valued roles in society, the psychological costs of not meeting narrow standards of success are reduced while allowing people to diversify their identities around contribution and meaning rather than social standing. People may then become more willing to explore career domains that are essential for societal functioning but frequently undervalued, such as care work, education, environmental stewardship, and community maintenance (Awang, 2022; E. Gould et al., 2021). In sum, increasing perceived affordances for status by broadening the activities that people can do to feel recognized and valued not only reduces mismatch-driven stress at the individual level, but also rebalances labor allocation toward broader societal needs at the level of the collective.

### 6.3. Encouraging In-Person Social Interactions

One of the most intuitive strategies to alleviate evolutionary mismatch is to encourage more physical, in-person social interactions. The very need for such a recommendation is itself a distinctly modern phenomenon because social life unfolded almost entirely face-to-face for most of human history, so physical co-presence required no deliberate effort. Only with the rise of digital communication and messaging platforms (e.g., social media, WhatsApp) has it become possible to maintain active social networks without interacting in person. This technological shift toward virtual connectivity satisfies some social needs but leaves evolved inclinations for embodied, face-to-face interaction unmet (Turkle, 2011). In addition, digitally mediated interactions remove constraints on aggressive or dominance signaling that would be naturally regulated in physically present settings (e.g., getting punched in the face for running one’s mouth), so hostility or posturing becomes especially rampant in online settings. Thus, explicitly encouraging people to seek out in-person contact has become necessary in a way that would have been strange in pre-digital times. Indeed, wellbeing often worsens when our actual experiences deviate from how we evolved to live in tight-knit and physically present groups (O et al., 2024), so restoring some degree of face-to-face interaction should yield benefits. Consistent with this, spending time in the company of family members (Savahl et al., 2023), friends (Van der Horst & Coffé, 2012), or even casual acquaintances is associated with improved wellbeing (Sandstrom & Dunn, 2014). These benefits can become especially important during periods of forced isolation, such as when there is severe atmospheric pollution (O & Liang, 2025) or during pandemics (Harriman et al., 2024).

It is important to acknowledge that virtual interactions can still provide meaningful socioemotional support, particularly when mobility is restricted. For instance, video-based communication where individuals can hear and see each other has been shown to sustain wellbeing during the COVID-19 pandemic (Dürst et al., 2022). Nonetheless, video interactions lack the tactile, embodied, and fully synchronous qualities of in-person encounters, and evidence suggests that face-to-face communication generally brings better socioemotional outcomes (Petrova & Schulz, 2022). Moreover, the widespread availability of online channels can cause people to default to these low-effort modes of communication instead of engaging in the more effortful process of meeting physically, resulting in the weakening of routines that promote offline social encounters (Turkle, 2011). Thus, in-person interactions are recommended, because although virtual communication is an important supplement—particularly when in-person contact is difficult—it displaces the richer forms of natural, real-life interaction that humans are adapted for.

### 6.4. Moderating Social Media Use

With mounting evidence that social media elevates competitiveness and stress in avid users, as well as health risks comparable to harmful substances (Haidt, 2024; Twenge et al., 2022), calls for regulatory measures have intensified in recent years (Office of the Surgeon General, 2023). Proposed regulations largely involve imposing caps on the consumption of (and time spent on) social media (Faulhaber et al., 2023; Root & Ashford, 2024), including bans on features that keep users constantly engaged (e.g., infinite scroll, autoplay; Social Media Addiction Reduction Technology Act, 2019). Australia has moved beyond feature-level restrictions and implemented one of the most stringent regulatory responses to date, namely a complete ban on social media use for under-16s, which has garnered mixed reactions from the public and sparked fears that youths may circumvent such restrictions through illicit workarounds or migrate toward less regulated and potentially more harmful online spaces (Pearlman, 2025).

Implicit in measures aimed at limiting social media use is the assumption that use itself is the primary cause of harm. Unlike harmful substances that can simply be avoided, however, social media has fundamentally reshaped how people access and exchange information in the digital age (Haidt, 2024). Hence, outright bans may be neither practical nor equitable, as such measures apply indiscriminately across users rather than selectively targeting harmful engagement (Hartanto et al., 2021). This approach further risks overlooking the benefits of social media, including its role in facilitating everyday communication and maintaining healthy social relationships—particularly among youths, for whom social interaction is increasingly organized around digital platforms (Vaingankar et al., 2022)—as well as its capacity to foster trust and engagement between brands and consumers (Labrecque, 2014). Social media also serves as a useful channel for learning about cultural and community events (e.g., music performances, art exhibitions), rendering complete disengagement wasteful. Importantly, the adverse effects of social media often arise not from use per se but from extensive exposure to more social information than people typically need, much of which originates from weak ties or strangers and often has questionable accuracy. Human psychological mechanisms designed to manage social information within small communities may be poorly equipped to evaluate such inputs, particularly among developmentally and dispositionally vulnerable users (Hartanto et al., 2021), thereby subjecting heavy users to unnecessary social comparisons and competitive pressures.

More nuanced strategies that mitigate harm while preserving the opportunities of social media are thus desirable. Accordingly, a preferable response to completely restricting use may be to recalibrate patterns of exposure toward smaller, evolutionarily familiar network sizes, rather than the evolutionarily novel, large-scale networks characteristic of contemporary platforms (A. J. Lim et al., 2021). Because social information from close ties is typically the most relevant, algorithms could be adjusted to prioritize content from strong social connections over that of weak ties or commercially driven sources, the latter of which are often optimized to exploit perceived deficiencies and insecurities in order to influence consumer behavior (Ekinci et al., 2025). In parallel, educational initiatives could increase awareness that social media content frequently presents a skewed representation of reality, while also emphasizing the importance of moderating reliance on social media and maintaining engagement in offline social interactions (Turkle, 2011). Together, these approaches would better align the flow of social and commercial information with the limits and capacities of evolved psychological mechanisms, thereby reducing unnecessary socioemotional influence without losing the benefits of social media.

### 6.5. Moderating Technological Use and Reliance

A crucial factor contributing to people leading increasingly mismatched lives is excessive reliance on technology, which results in less natural, original, or direct ways of engaging with the world (Yong & Kanazawa, 2026). For instance, transport technologies allow us to use less effort and reach destinations faster. However, they replace walking or running to get around, which comes with benefits such as metabolic regulation and greater connectedness with the environment. Similarly, entertainment technologies like computer games and on-demand streaming services have supplanted simpler pastimes like reading, playing outdoors, or engaging in hands-on hobbies (e.g., crafting, building). Overreliance on technology can also undermine the development and upkeep of useful competencies. For example, once calculators became widespread, fewer people practiced manual calculations; likewise, once navigational apps (e.g., Google Maps) came about, people increasingly experience difficulties finding their way around without digital guidance (Dahmani & Bohbot, 2020). Modern technologies are also reshaping core human experiences in ways that undermine well-being. For instance, people now have the option of choosing virtual reality experiences over spending time in nature (Michaelson et al., 2020), or pornography and sex robots over real sexual relationships (Brooks, 2021). These shifts carry real costs as they prevent us from appropriately stimulating the biological, cognitive, and social adaptations that support optimal functioning and well-being (Yong & Kanazawa, 2026).

Therefore, reducing technology usage, even temporarily or by choice rather than necessity, can reap a range of positive outcomes. Engaging in less technologically mediated actions like walking to the store instead of driving, cooking meals instead of ordering delivery, spending time outdoors without screens, navigating without GPS, or building something by hand instead of relying on pre-made solutions can cultivate mindfulness, strengthen problem-solving skills, promote physical movement and sensory engagement, and foster a sense of accomplishment (Boere et al., 2023; Collier et al., 2020; Farmer & Cotter, 2021). Educational campaigns can further support these efforts by promoting consumer literacy and emphasizing that the benefits of technology depend on actual utility rather than trends, novelty, or marketed upgrades. Importantly, not all technologies need to be reduced; essentials such as correction glasses for individuals with myopia, medication when ill, or mobile phones for everyday communication remain necessary. The key challenge is determining a realistic level of reduction, which in turn can reduce unnecessary technological immersion by highlighting situations where less tech-intensive alternatives suffice (Yong & Kanazawa, 2026).

The growing integration of artificial intelligence (AI) across key domains of human activity warrants special attention. While AI provides advantages such as greater access to information and increased analytical efficiency, it also raises challenges, including job insecurity, difficulty discerning the credibility of content, and the proliferation of deepfakes (A. J. Lim et al., 2026b). Moreover, depending too much on AI can reduce the need for cognitive effort and impair our ability to think critically (Wiederhold, 2025). Students may default to using AI for assignments instead of engaging deeply with material, and workers may lean on AI solutions rather than building domain expertise and experiential competence. The rise of AI-generated academic papers with made-up data and references further highlights the risks to scholarly integrity (Spinellis, 2025). However, fully eliminating AI is neither realistic nor desirable given its rapid diffusion and embeddedness in everyday cognitive and institutional practices. A more constructive approach is to recognize its inevitability and benefits while intentionally moderating usage in order to preserve the things that matter, such as independent reasoning, creativity, and authenticity (Wiederhold, 2025). Setting boundaries such as using AI only as a supplementary tool, withholding its use for tasks that require deep understanding, and consciously practicing independent problem-solving can help maintain cognitive engagement and skill development. By understanding both its pros and cons, AI can be utilized in ways that enhance human capabilities while minimizing dependency, cognitive atrophy, and the fading sense that a human mind stands behind the work.

### 6.6. Increasing Engagement with Nature

Engaging with nature can help rebuild biopsychosocial resources that modern environments often erode. A wealth of research has shown that nature contact decreases physiological stress markers (Yao et al., 2021), enhances positive affect (McMahan & Estes, 2015), reduces depressive mood (Roberts et al., 2019), and increases prosocial behavior (Arbuthnott, 2023). One mechanism through which immersion in nature produces these benefits is increased exposure to phytoncides, which have positive psychophysiological effects (Lew & Fleming, 2024), and to diverse environmental microbes that can beneficially shape our microbiome (Nurminen et al., 2018). These exposure benefits can accumulate into enduring improvements in health and wellbeing as nature contact has been linked to stronger anti-inflammatory responses and natural killer cell activity, indicating enhanced immune functioning (Andersen et al., 2021), as well as quicker and fuller recovery following adversity (White et al., 2019). Yet urbanization and digitalization have reduced outdoor experiences and increased time spent on screens, creating a disconnect that weakens people’s relationship with the natural world (Beery et al., 2023; Soga & Gaston, 2016). Rebuilding a sense of connectedness with nature—not merely facilitating physical exposure—is therefore beneficial. Connectedness to nature predicts happiness (Capaldi et al., 2014) and eudaimonic wellbeing (Pritchard et al., 2020) and helps transform occasional nature exposure into enduring engagement (Sheffield et al., 2022), creating a reinforcing cycle of seeking and benefitting from nature.

It is important to note that nature is not uniformly or automatically experienced as positive. People who grew up in highly urbanized contexts typically have limited exposure to nature and while they report comparable levels of positive affect in both natural and built green spaces, they can experience greater apprehension in more naturalistic environments because these are perceived as “wilder” and less safe (Dillon et al., 2024). Thus, built green spaces (e.g., parks, streetscapes, curated greenery in buildings) serve as intermediary environments that provide the benefits of nature while eliciting lower levels of apprehension. Residential proximity to greenspace is associated with lower cardio-metabolic risks and disease mortality (Gascon et al., 2016; Kardan et al., 2015), lower likelihood of developing psychiatric disorders (Engemann et al., 2019), and greater neighborhood cohesion through improved social ties, place attachment, and trust (Arbuthnott, 2023).

Ways to increase engagement with nature vary across the lifespan. In childhood, regular unstructured outdoor play or gardening programs in schools can establish comfort with and curiosity toward the natural world (Kong & Chen, 2024; Skalstad & Munkebye, 2022). For adults, especially those who grew up without much nature exposure and may have developed some degree of biophobia, self-paced or guided activities such as neighborhood walks or nature-based mindfulness can help re-establish confidence and positive associations with nature (Barragan-Jason et al., 2022). Older adults may benefit from socially oriented and low-intensity activities in nearby green spaces which support both mobility and social connection (M. Tan et al., 2023). These activities could be further enhanced through intergenerational engagement such as those that take place in community gardens (Koay & Dillon, 2020).

### 6.7. Encouraging Natural Diets

Given the potential harms that highly processed foods can pose to wellbeing, it is unsurprising that empirical evidence favors diets comprising “minimally processed foods close to nature” (Katz & Meller, 2014, p. 83). These include vegetables (especially non-starchy vegetables; e.g., leafy greens, cruciferous vegetables), intact fruits with natural fiber, legumes (e.g., beans, lentils, chickpeas), intact or minimally milled (i.e., whole) grains, nuts and seeds (e.g., almonds, sesame), fish and seafood (especially in Mediterranean patterns), and lean, minimally treated animal foods (e.g., lean meats, eggs), preferably unsalted and unrefined (Cena & Calder, 2020; Krznarić et al., 2021; LeBlanc et al., 2024). Such foods are considered beneficial because they align more closely with what our physiological and metabolic systems evolved to process and serve as healthier alternatives to the mass-produced, highly processed, and artificial consumables sold in supermarkets and convenience stores today.

To prompt people to eat natural foods more, various strategies have been proposed. Approaches that emphasize informed, volitional decision-making typically involve health promotion efforts that provide guidance on healthier food options or encourage individuals to reflect on their health status (e.g., biometric feedback devices, free health-screening services), and these have been shown to improve dietary habits (Abril & Dempsey, 2019). However, educational or persuasive methods may not always work as people often face practical barriers, such as limited availability of healthy options or lifestyle constraints (e.g., work routines, costs). In such cases, interventions that modify affordances for healthier diets like coupons for disadvantaged populations, subsidies or levies to incentivize healthier choices, serving healthier meals in educational and workplace settings, or enforcing mandatory standards for healthy eating represent viable alternatives (Brambila-Macias et al., 2011).

### 6.8. Moderating Consumption

Having the urge to acquire, stockpile, and consume resources was adaptive in ancestral contexts characterized by uncertainty and scarcity. In contrast, modern environments provide relatively stable and abundant access to goods and services, yet the adaptations that motivate consumption continue operating. This is compounded by highly targeted advertising (Nyrhinen et al., 2024; Pellegrino et al., 2022) and socially competitive environments that frame consumption as a signal of status and belonging (Pandelaere, 2022; Yong et al., 2024). When left unchecked, consumption can become less about meeting adaptive needs and more about status competition, which can increasingly preoccupy people’s attention and contribute to materialism, chronic comparison, status anxiety, and reduced investment into other important adaptive domains (N. P. Li et al., 2015).

From our perspective, moderating consumption can be understood as a process of recalibrating behavior to current conditions and sufficiency. Effective recalibration is unlikely to arise from “buy less” advice alone and would instead benefit from supports that shift default decisions, such as norms and incentives that make moderated consumption, delayed gratification, and sufficiency-oriented practices easier to adopt in everyday life (Ganglmair-Wooliscroft & Wooliscroft, 2025). Consumer literacy initiatives that raise awareness of how modern resource availability reduces the necessity of stockpiling, how advertising exploits evolved acquisition tendencies (Danciu, 2014; Ekinci et al., 2025), and the financial and psychosocial consequences of overconsumption may further support individuals in disengaging from externally induced desires and regulating consumption behavior (Iniesta-Bonillo et al., 2025; Muñoz-Céspedes et al., 2021). Importantly, reducing consumption does not imply deprivation; rather, it can allow individuals to redirect attention and effort toward other domains that are less dependent on material accumulation, such as social relationships, skill development, and engagement with the natural world. Indeed, the emerging popularity of movements and philosophies such as minimalism (Conroy, 2020), decluttering (Kondo, 2014), and voluntary simplicity lifestyles (Osikominu & Bocken, 2020) reflects a growing collective recognition of the benefits of a less materialistic and consumption-intensive way of life.

### 6.9. Operationalizing and Evaluating Mismatch-Reduction Interventions

To evaluate the effectiveness of interventions, those for reducing social evolutionary mismatch can first be specified in terms of the mechanisms they are targeting (i.e., scale calibration, signal validity, exposure duration) and then operationalized in terms of changes in exposure, frequency, intensity, or subjective experience. For example, moderating consumption can be operationalized as reductions in the frequency or quantity of discretionary purchases, thus reflecting changes in signal validity such as reduced reliance on exaggerated or low-diagnostic status cues. Some mismatches may be more meaningfully captured at the level of individual perception rather than objective conditions. For instance, population density may not uniformly predict psychological outcomes unless it is experienced as crowded or competitive (E. Tan et al., under review), thus underscoring the importance of subjective appraisal in shaping scale calibration processes. Accordingly, mismatch reduction can also be operationalized as perceived changes in crowdedness, competitiveness, or availability of social support. The evolutionarily mismatched lifestyle scale (O et al., 2024), which assesses individual differences in exposure to mismatches across domains such as diet, physical activity, and social media use, is particularly valuable for capturing the subjective experience of evolutionary mismatch directly.

For non-social interventions, reducing technological reliance can, for instance, be operationalized in terms of behavioral substitution and effort engagement, such as the extent to which individuals opt for technology-mediated convenience (e.g., driving, using the elevator, food delivery) over more effortful alternatives (e.g., walking, using the stairs, going out to eat or making a meal from scratch), as well as overall levels of physical activity and engagement in effortful tasks. Increasing engagement with nature can be quantified as time spent in natural environments or amount of greenness in one’s home or workplace, which is in line with aligning with evolutionarily familiar inputs.

Evaluation should then draw on converging indicators that capture the effects of mismatch reduction. These include physiological markers such as salivary cortisol, heart rate variability, blood pressure, and neural responses to stress (Evans & Wener, 2007; Lederbogen et al., 2011; A. J. Lim et al., 2026a), subjective measures like the positive and negative affect schedule (Watson et al., 1988) and the satisfaction with life scale (Diener et al., 1985), and behavioral outcomes including cooperation (L. K. Tan et al., 2024), aggression (Xie et al., 2025), and prosociality (Choy et al., 2021), alongside shifts in attention allocation (e.g., reduced focus on status-relevant information) and everyday activity patterns (e.g., daily diary studies). These indicators can then be examined in relation to changes in mismatch reduction to assess whether interventions are associated with improvements in physiological and psychosocial functioning. Evaluation efforts can be complemented with experiments, which can isolate specific components of mismatch by manipulating environmental inputs (e.g., exposure to natural vs. urban settings, high vs. low visibility of status cues), and comparative ecological analyses, which can examine how naturally occurring variation in mismatch relates to outcomes across populations (e.g., people living in rural vs urban places, citizens of high vs low GINI countries). Although these approaches do not directly evaluate interventions, they help establish causal mechanisms and boundary conditions that inform the design and interpretation of mismatch reduction strategies.

Existing research provides some preliminary evidence that individuals residing in less mismatched environments generally experience lower levels of deleterious indicators (Lederbogen et al., 2011; A. J. Lim et al., 2026a; O et al., 2024). For example, perceptions of lower crowdedness are associated with reduced cortisol levels (Evans & Wener, 2007), aggressive behavior (Xie et al., 2025), and competitiveness (E. Tan et al., under review), which also highlights the role of subjective experience in mediating the effects of environmental conditions. Experimental studies manipulating the degree of built features in the environment also revealed that participants exposed to less built features experienced lower stress, more positive mood, and better workplace functioning including reduced emotional exhaustion and interpersonal conflict (A. J. Lim et al., 2026a). More targeted evaluations are needed to assess mismatch reduction in other domains, particularly those involving technological reliance, consumption patterns, and the expansion of meaningful and socially valued roles.

It is important to note that quantifying the benefits of mismatch reduction is not always straightforward. Strict definitions of evolutionary mismatch emphasize that “evidence that the reproductive fitness or well-being of individuals is negatively impacted […] is not necessary to substantiate input-driven mismatch” (N. P. Li et al., 2018, p. 40), because evolutionarily novel inputs (e.g., technology, complex social organization) *can* provide immediate benefits by addressing needs or solving problems, even as they introduce longer-term misalignments and problems (Yong & Kanazawa, 2026). This presents a challenge because reducing exposure to such inputs may not produce immediate improvements and may even be experienced as aversive in the short term. For example, moderating consumption, limiting social media use, or shifting away from highly palatable foods may involve discomfort or perceived loss despite their potential longer-term benefits. Thus, evaluations of mismatch reduction strategies should not rely solely on immediate subjective responses, but instead consider their impact on underlying input mechanisms (e.g., scale calibration, signal validity, exposure duration) and longer-term patterns of behavior, attention allocation, and functioning across domains. In some cases, interventions that reduce mismatch may initially worsen subjective experience while improving more hidden regulatory processes. A proper evaluation of intervention effectiveness therefore requires a deep appreciation of how evolved adaptations work, and that restoring alignment between the environment and our adaptations may involve short-term costs alongside more durable improvements in regulation, social dynamics, and overall well-being.

## 7. Discussion

In this paper, we highlighted how misalignments between adaptive mechanisms and modern environments intensified by the convergence of large-scale crises (e.g., climate change, geopolitical conflict, technological disruption, economic volatility) create conditions that give rise to interconnected psychosocial problems (e.g., anxiety, relationship difficulties, cynicism, depression). Building on these foundations, we introduced the SEMCH, proposing that social forms of mismatch (e.g., burgeoning population sizes and peer networks, weak ties and fragmented communities, socioeconomic inequality) uniquely amplify both real and perceived competition for modern humans. This perspective helps account for diverse behavioral outcomes, including obsessive status pursuit, hostility, and social withdrawal, and enable these phenomena to be understood not just as cultural or economic reactions, but as deep-seated responses to evolutionarily novel levels of intensified competition. As such, we demonstrate how evolutionary mismatch can serve as a potent theoretical framework that weaves together disparate modern challenges, helping to clarify the dynamics of the contemporary polycrisis and why everyday social life is so widely experienced as relentlessly competitive and stressful.

The present analysis also yields important practical implications. We outlined interventions that directly address sources of evolutionary mismatch, which converge with a body of practical advice emphasizing lifestyle simplification, consumption reduction, and reconnection with natural processes (Barragan-Jason et al., 2022; Conroy, 2020; Osikominu & Bocken, 2020), while tempering optimism on techno-solutionist approaches to manage the rising tide of modern problems (Yong & Kanazawa, 2026). Indeed, although technological innovations can sometimes provide substantial benefits (e.g., revolutionary technologies that lead to breakthroughs in energy production or life sustenance), they also risk intensifying evolutionary mismatch by further distancing human environments from natural conditions. Moreover, many technologies provide narrow, downstream fixes, whereas mismatch-reduction approaches operate at a more fundamental level, simultaneously alleviating multiple sources of mismatch while generating fewer unintended side effects (N. P. Li et al., 2020).

Finally, the evolutionary mismatch perspective adds a crucial applied dimension to evolutionary psychological and behavioral science, which has been criticized as relying on untestable “just-so stories” (S. J. Gould & Lewontin, 1979). By making these ideas relevant to real-world challenges (N. P. Li et al., 2020), policymakers and industry practitioners may have a clearer understanding of how to adopt and apply them, allowing their effectiveness to be observed in practice and thereby helping to validate evolutionary theories while narrowing the divide between theory and practice.

### 7.1. Caveats, Limitations, and Future Directions

In the interest of brevity, we confined our discussion of adaptive needs to several well-known ones, though others could also plausibly be included. For instance, parenting is an important domain that was not discussed. Nevertheless, unlike other adaptive needs like belonging or mating, which are associated with motives to be socially included or to attract mates, parenting does not necessarily arise from a direct motive to become a parent. For many individuals, children are the consequence (or even byproduct) of mating and reproductive motives, after which parenting emerges as an adaptive problem that must then be dealt with. With that said, the present framework can still be applied to the parenting domain to illuminate potential evolutionary mismatches there. For example, in ancestral environments, the burden of childrearing was typically shared across multiple caregivers, whereas parents in modern societies often raise children with significantly less support (Chaudhary & Swanepoel, 2023). In addition, ancestral parents and children were embedded in environments that changed little between generations, whereas contemporary societies undergo such rapid cultural and technological shifts that the world can differ tremendously across a single generation. This accelerates intergenerational divergence and makes it harder for parents to remain relevant or effective sources of guidance. The present paper therefore provides a general framework for analyzing any adaptive domain in terms of potential evolutionary mismatches in modern environments.

While the mismatch framework offers a unifying account of diverse behaviors ranging from heightened competitiveness to disengagement, this breadth also raises its risk of becoming a “theory of everything” that explains nothing with precision and is difficult to test or falsify. We have attempted to address this by deriving a number of precise testable predictions from the SEMCH and introduced perceived surmountability, autonomy, fairness, and cultural context as moderators of its predicted downstream consequences. However, these moderators were not discussed at length, and we wish to be clear that evolutionary mismatch should not be viewed as the sole driver of all modern problems. Other more proximate cultural, institutional, economic, and personality influences play critical roles in determining the extent to which evolutionary mismatch impacts modern humans and require deeper elaboration and empirical testing.

A critical nuance to be aware of is that evolutionary mismatch should be strictly defined by how evolutionarily novel environments interfere with adaptive function, rather than just by decrements in well-being, health, or reproductive success (N. P. Li et al., 2018). This is important for two reasons. First, to make our point about how evolutionary mismatch contributes to modern problems, we mainly presented cases where modern environments are associated with deleterious outcomes, while the other reality—that people are also capable of thriving in highly urbanized, evolutionarily novel environments that have brought benefits including reduced violence, increased longevity, and expanded access to knowledge—was mostly left out. Indeed, evolutionarily novel inputs can support fitness-related outcomes. For example, individuals can avoid starving to death by overconsuming unhealthy foods because they are still able to secure calories. As this shows, it can be misleading to assess evolutionary mismatch on the basis of simple fitness metrics like longevity alone, and it is necessary to consider both adaptation–environment alignment as well as other problems that later emerge. Conditions that improve some aspects of survival may simultaneously create or worsen other adaptive challenges. For instance, extended lifespans are associated with age-related disease, loneliness, and dependence on medical interventions to stay alive (Yong & Kanazawa, 2026). Thus, observing that people can still do reasonably well on some fitness metrics in evolutionarily mismatched modern contexts does not detract from our overall point that evolutionary mismatch has a significant hand in modern problems. Success within modern environments may coexist with trade-offs that are not immediately apparent, so a more comprehensive analysis of evolutionary mismatch requires consideration of these trade-offs, including the secondary problems introduced by evolutionarily novel forms of comfort and convenience.

A second, related caveat that follows is that we are not suggesting that all modern technologies are inherently bad. Many modern technologies (e.g., antibiotics, electric heating, water sanitation) allow us to survive where our ancestors could not, and even more debatable technologies like social media have demonstrated both negative and positive effects, such as supporting social needs when more traditional forms of contact are not available (Szeto et al., 2024). Technologies are after all created to address unmet adaptive needs, so they usually have at least some degree of usefulness despite being evolutionarily novel. However, the very conditions that give rise to technological usefulness also set the stage for evolutionary mismatches, as many contemporary problems are themselves downstream consequences of prior technological reliance (Yong & Kanazawa, 2026). New technologies introduced to address problems may also sometimes be already deeply enmeshed in mismatch, such as the rise of AI-powered entities that people can form parasocial relationships with to deal with loneliness or anxiety (Gregg, 2025). The central question, then, is not whether technology should be used—it should, because it *does* have utility—but rather how its use can be regulated to address genuine needs without making us worse off. The broader aim of the interventions we proposed is to suggest ways to achieve this balance by selectively reducing or modifying specific sources of evolutionary mismatch, particularly those that are feasible to implement and desirable to change, while preserving the functional advantages of modern systems. Achieving this requires a keen understanding of what is beneficial for humans in general, as well as one’s own adaptive needs more specifically.

Despite the clear value of the present framework as a means for deeper analysis of modern problems, it is still necessary to exercise some level of caution against overreaching the impact of evolutionary mismatch. For instance, the degree to which psychosocial phenomena are due to mismatch—from psychological conditions like attention-deficit/hyperactivity disorder to social behaviors like outgroup hostility—have been contested (Dein, 2024; Hoogland & Ploeger, 2022; Segovia-Cuéllar & Del Savio, 2021). Our aim in this paper is not to adjudicate these debates but to illustrate the breadth of phenomena that have been examined through a mismatch lens, and we encourage readers to engage with these discussions more closely. In addition, reducing evolutionary mismatch may not necessarily yield benefits across all types of outcomes. For example, a series of experiments testing whether exposure to more evolutionarily familiar (i.e., less mismatched) environments would enhance creativity found that although the manipulation produced expected gains in physiological and affective outcomes, creative performance did not improve (A. J. Lim et al., 2026a). Thus, mismatch reduction may not uniformly enhance all outcomes, particularly those that are context-dependent (creativity, for example, may be selectively expressed in environments where novel problem-solving is functionally relevant, rather than in decontextualized laboratory tasks) or less critical to survival. Further studies can seek to identify which classes of outcomes are most likely to benefit from mismatch reduction, with particular attention to the boundary conditions in which evolutionary mismatch may exert effects.

### 7.2. Conclusions

In summary, we have shown how the multiple crises affecting modern humans can be understood through an evolutionary mismatch framework, highlighting both the consequences that arise when our evolved adaptations are misaligned with contemporary environments and the mismatch-informed solutions that may help mitigate them. While many of the individual examples and topics discussed are well established within their respective fields, this paper demonstrates how they can be integrated under a unifying framework, revealing a common thread that links diverse phenomena. Future tests of the assumptions and hypotheses presented here are welcome and likely to engender critical insights into addressing the urgent challenges facing society today.

## Figures and Tables

**Table 1:** Components of the contemporary polycrisis.

Problem	Examples
Climate and ecological crisis	Global warming Biodiversity loss Deforestation, ocean acidification
Energy and resource constraints	Energy price volatility Critical mineral shortages Water scarcity
Food system instability	Supply chain fragility Climate impacts on agriculture Fertilizer and input shocks
Economic and financial instability	Inflation, debt crises Wealth inequality Cost-of-living crises
Geopolitical conflict	Great power rivalry Wars and regional conflicts Trade disruptions and sanctions
Public health crises	Pandemics Antimicrobial resistance Strained healthcare systems
Information and institutional breakdown	Misinformation/disinformation Declining trust in institutions Political polarization
Technological disruption	AI and automation shocks Cybersecurity risks Surveillance vs privacy tensions
Social fragmentation	Cultural polarization Migration pressures Ethnic tensions

**Table 2:** Sources of evolutionary mismatch due to differences between ancestral and modern environments.

	Ancestral Environment	Modern Environment
Population size	~150	Hundreds of thousands; crowded
Environmental features	Natural; e.g., foliage, savannah	Urban; e.g., concrete buildings, cemented roads
Societal structure	Egalitarian	High inequality
Lifestyle	Nomadic; natural diets	Sedentary; artificial diets
Economic arrangement	Primitive; barter trade	Advanced; capitalistic
Technological penetration	Basic; simple tools	Advanced; e.g., cars, computers
Informational complexity	Low	High; e.g., mass media, information technology, social media
Social complexity	Low	High; e.g., complex hierarchies
Environmental stability and risk	Stable, slow-changing; recurrent, isolated, and predictable threats	Rapidly shifting; unpredictable and globally entangled threats

## Data Availability

No new data were created or analyzed in this study.
